# Mechanomyographic amplitude and frequency responses during dynamic muscle actions: a comprehensive review

**DOI:** 10.1186/1475-925X-4-67

**Published:** 2005-12-19

**Authors:** Travis W Beck, Terry J Housh, Joel T Cramer, Joseph P Weir, Glen O Johnson, Jared W Coburn, Moh H Malek, Michelle Mielke

**Affiliations:** 1 Department of Nutrition and Health Sciences, Human Performance Laboratory, University of Nebraska-Lincoln, Lincoln, NE, USA 68583; 2 Department of Health and Exercise Science, University of Oklahoma, Norman, OK, USA 73019; 3 Applied Physiology Laboratory, Division of Physical Therapy, Des Moines University, Osteopathic Medical Center, Des Moines, IA, USA 50312; 4 Department of Kinesiology, California State University, Fullerton, Fullerton, CA, USA 92834

## Abstract

The purpose of this review is to examine the literature that has investigated mechanomyographic (MMG) amplitude and frequency responses during dynamic muscle actions. To date, the majority of MMG research has focused on isometric muscle actions. Recent studies, however, have examined the MMG time and/or frequency domain responses during various types of dynamic activities, including dynamic constant external resistance (DCER) and isokinetic muscle actions, as well as cycle ergometry. Despite the potential influences of factors such as changes in muscle length and the thickness of the tissue between the muscle and the MMG sensor, there is convincing evidence that during dynamic muscle actions, the MMG signal provides valid information regarding muscle function. This argument is supported by consistencies in the MMG literature, such as the close relationship between MMG amplitude and power output and a linear increase in MMG amplitude with concentric torque production. There are still many issues, however, that have yet to be resolved, and the literature base for MMG during both dynamic and isometric muscle actions is far from complete. Thus, it is important to investigate the unique applications of MMG amplitude and frequency responses with different experimental designs/methodologies to continually reassess the uses/limitations of MMG.

## Background

**Figure 1:**
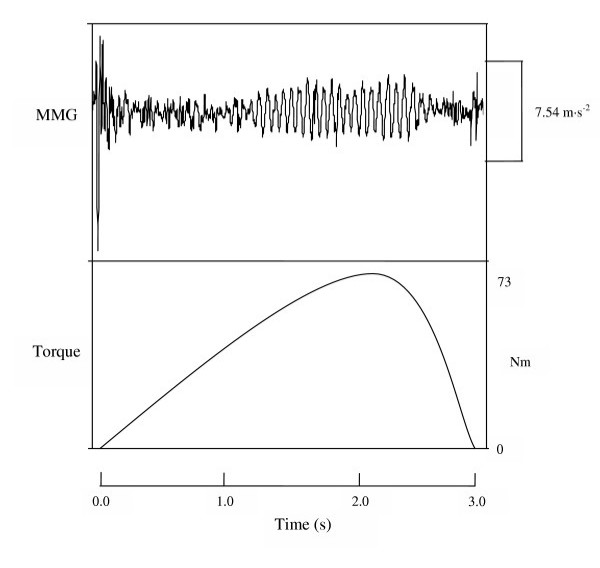
**Mechanomyographic (MMG) signal during a voluntary muscle action**. Example of the raw mechanomyographic (MMG) signal from the biceps brachii muscle and the torque production curve during a concentric isokinetic muscle action of the forearm flexors at 100% peak torque (PT) for one subject. The concentric muscle action was performed at a velocity of 30°·s^-1 ^across a 90° range of motion (180° to 90° of forearm flexion), and the MMG signal was recorded with an accelerometer (Entran EGAS FT 10).

Mechanomyography (MMG) involves recording and quantifying the low frequency lateral oscillations of active skeletal muscle fibers [1,2]. Although the exact origin(s) of the MMG signal is not completely understood, Gordon and Holbourn [3] suggested that MMG reflects the mechanical counterpart of motor unit electrical activity as measured by electromyography (EMG). During voluntary muscle actions, MMG is usually measured at the surface of the skin over a muscle, and it has been suggested [1,4-6] that in this situation, the MMG signal is generated by three primary mechanisms: a) gross lateral movements of the muscle as it moves toward, or away from, its line of pull during contraction and relaxation, respectively, b) smaller subsequent lateral oscillations of the muscle at its resonant frequency, and c) dimensional changes of the active fibers (see Figure 1).

Although various terminologies, such as soundmyography, phonomyography, acousticmyograpy, and vibromyography have been used by previous studies to describe the MMG signal, Orizio [4] recommended that the term "mechanomyogram" should be used to reflect the mechanical nature of the signal. Many different types of sensors have been used to detect the MMG signal, including hydrophones, condenser microphones, piezoelectric contact sensors, accelerometers, and, more recently, laser distance sensors [4,5]. As acknowledged by Orizio [4], the most important characteristic of the sensor is its frequency response. Specifically, Orizio [4] recommended that the "...low frequency cut-off has to be around 1 to 2 Hz," and "the upper cut-off has to be chosen so that the greater part of the power is well below 100 Hz." Another important characteristic is the mass of the sensor. In particular, it has been suggested [4] that lightweight accelerometers may be more appropriate than larger condenser microphones and piezoelectric contact sensors when recording MMG signals from small muscles, such as the first dorsal interosseous. For larger limb muscles, such as the biceps brachii and superficial quadriceps femoris muscles, however, condenser microphones and piezoelectric contact sensors can be used, as long as the contact pressure of the sensor over the muscle remains constant [4,7]. Although the shape of the MMG signal is dependent on the type of sensor used to detect it [4], Orizio et al. [5] have suggested that its pattern is similar to the small oscillations in force that occur during an isometric muscle action. Furthermore, it has been hypothesized [4,8-11] that both signals (MMG and force) contain information regarding motor control strategies (relative contributions of recruitment and firing rate). In particular, several investigations [5,8,10,12-14] have suggested that under certain conditions, the amplitude of the MMG signal may be related to the number of active motor units (i.e. motor unit recruitment), while the MMG power density spectrum could provide qualitative information regarding the global firing rates of the unfused activated motor units.

Most studies have examined the MMG amplitude and/or frequency responses during isometric muscle actions [15-20]. Stokes [6] suggested, however, that "If AMG [MMG] is to be used as a means of monitoring force during functional activities, then its relationship with force during dynamic activation must be considered." In addition to the implications related to monitoring functional and/or sporting activities, MMG responses during dynamic muscle actions may be particularly important for clinical applications such as controlling external prostheses [21], assessing low back pain which can be exacerbated during movement [22], monitoring rehabilitation following injury [4,23], and examining masseter muscle function associated with cranio-mandibular disorders [24]. Unfortunately, there are a number of factors that can potentially affect the MMG signal during dynamic muscle actions, including changes in torque production, muscle length, and the thickness of the tissue between the muscle and the MMG sensor [4,25]. Theoretically, one or more of these factors can influence the amplitude and frequency of the MMG signal during a dynamic muscle action and confound the interpretation of the underlying motor control strategies. There are, however, several pieces of evidence that indicate that the MMG signal is generated primarily by muscle activity during a dynamic muscle action. Briefly, MMG amplitude increases with torque production during concentric and eccentric muscle actions [26-28], as well as with increases in power output during incremental cycle ergometry [29-32]. These responses suggest that MMG may provide information regarding the level of muscle activity that is required to perform an exercise task. In addition, for a given torque level, MMG amplitude is less during eccentric muscle actions than concentric muscle actions [26], which is consistent with EMG data showing that a lower level of muscle activity is required during eccentric activity [33,34]. Shinohara et al. [30], however, provided perhaps the most convincing evidence that MMG reflects muscle activity during dynamic muscle actions. The authors [30] examined the MMG amplitude responses for the vastus lateralis muscle during an incremental cycle ergometry test in which the power output was increased by 20 W each minute and the subject maintained a pedal cadence of 60 revolutions per minute. During the first 30 s of each 1-minute stage, the subjects were instructed to relax with their feet on the pedals while one of the investigators pushed the pedals at the required cadence. For the second 30 s of each one minute stage, however, the subjects pedaled actively against the ergometer. The authors [30] reported that MMG amplitude for the vastus lateralis muscle increased linearly with power output during the active cycling, but there was only a small increase in MMG amplitude during the passive cycling. In addition, it was hypothesized that the small increases in MMG amplitude during the passive cycling may have been due to incomplete relaxation in some of the subjects, because there was also a small increase in EMG amplitude for the vastus lateralis muscle. Thus, the authors [30] stated, "These observations confirm that the MMG recorded during the contraction phase could include some noise, but mainly reflect the activity of the quadriceps muscle within the parameters of this experiment." These findings [26-28,30-32] clearly indicated that during dynamic muscle actions, MMG is generated primarily by muscle activity. Therefore, pertinent follow-up questions include: a) what kind of information can be extracted from the MMG signal that is recorded during a dynamic muscle action; and b) what are the uses/applications of this information? Unfortunately, there are no simple answers to these questions because the MMG amplitude and frequency responses depend largely on the type of dynamic muscle action(s) (i.e. concentric versus eccentric, maximal versus submaximal) that is being performed. Furthermore, there are many issues regarding the exact origin(s) of the MMG signal that have yet to be resolved. The purpose of this review, however, is to examine the literature regarding the MMG responses during different types of dynamic muscle actions. Although many of the studies discussed in this review are contributions from our laboratory, an attempt has been made to be as comprehensive as possible, and emphasis will be placed on summarizing the common findings from the various types of experiments that have been performed.

## MMG amplitude and frequency responses with increases in velocity during maximal concentric isokinetic muscle actions

Evetovich et al. [35] were the first to examine the MMG amplitude responses with increases in velocity during isokinetic muscle actions. The authors [35] reported that during maximal concentric isokinetic leg extensions, there was a velocity-related increase in MMG amplitude for the vastus lateralis muscle, but leg extension peak torque (PT) decreased with increases in velocity from 60–360°·s^-1^. In addition, the MMG amplitude values were highly reliable, with intraclass correlation coefficients (ICCs) ranging from R = 0.90–0.99, with no significant differences between the mean MMG amplitude values for test versus retest at any velocity. Evetovich et al. [35] hypothesized that the increase in MMG amplitude for the vastus lateralis muscle with an increase in velocity may have been due to reduced muscle stiffness at the high velocities. Specifically, at slow velocities, both slow- and fast-twitch muscle fibers contribute to torque production [35,36]. With increases in velocity, however, slow-twitch muscle fibers may become unloaded, because they are unable to contract rapidly enough to keep up with the speed of the movement [36]. As a result, there are decreases in PT and muscle stiffness, which may result in increased muscle fiber oscillations and greater MMG amplitude values [35]. Smith et al. [37] provided support for this hypothesis by demonstrating that during maximal concentric isokinetic muscle actions of the forearm flexors at velocities ranging from 30–150°·s^-1^, there was a velocity-related increase in MMG amplitude for the biceps brachii, but forearm flexion PT decreased with increases in velocity. Furthermore, Evetovich et al. [38] found that during maximal concentric isokinetic leg extensions at velocities ranging from 30–150°·s^-1^, there were velocity-related increases in MMG amplitude for the vastus lateralis muscle in both men and women subjects. In addition to decreases in muscle stiffness, the authors [38] hypothesized that the increases in MMG amplitude at high velocities could be due to factors such as: a) a greater rate of cross-bridge cycling at high velocities that caused larger vibratory motions of the sarcomeres, and/or b) greater intracellular and extracellular fluid turbulence. The velocity-related increases in MMG amplitude were greater, however, for the men than for the women, and the men demonstrated greater MMG amplitude values than the women at all velocities [38]. It was suggested that the gender differences in the MMG amplitude patterns could be related to a greater decline in leg extension PT with increases in velocity for the women (33.3% decline) than for the men (28.5% decline). In addition, the greater MMG amplitude values for the men may have reflected differences in fiber type composition [39], a larger muscle mass, and/or a thinner adipose tissue layer over the vastus lateralis than is typically found in women [38]. In particular, English et al. [39] recently reported that the masseter muscle in male rabbits contained more fast-twitch fibers and fewer slow-twitch fibers than the same muscle in female rabbits. If these differences also occur in large limb muscles of humans, then gender differences in the MMG amplitude and/or MPF responses could be related to unique muscle fiber type distribution patterns in men versus women.

Theoretically, a velocity-related shift in the contributions of slow-twitch muscle fibers to torque production could also influence the frequency content of the surface MMG signal. In particular, if slow-twitch muscle fibers become unloaded at high velocities, it is possible that their contribution to MMG decreases. Several studies [40-42] have reported that when compared to low threshold slow-twitch motor units, high threshold fast-twitch motor units have higher initial firing rates and require greater stimulation rates to achieve complete fusion of motor unit twitches. Thus, decreases in the contributions of slow-twitch muscle fibers to MMG at high velocities could result in higher frequency MMG signals. Ebersole et al. [43] tested this hypothesis by examining the MMG amplitude and mean power frequency (MPF) responses from the vastus lateralis muscle during maximal concentric isokinetic leg extensions at velocities of 60 and 300°·s^-1^. The authors [43] reported that with an increase in velocity from 60 to 300°·s^-1^, there was a significant decrease in leg extension PT and an increase in MMG amplitude. There was, however, no difference between the mean MMG MPF values at 60 and 300°·s^-1^. It was hypothesized that the tendonous iliotibial band that covers the vastus lateralis muscle could have interfered with the muscle fiber oscillations that generated the MMG signal. In addition, it was still unclear if the velocity-related increases in MMG amplitude that had been reported for the vastus lateralis [35,38,43] and biceps brachii [37] were due to reduced muscle stiffness, a greater rate of cross-bridge cycling, or increased turbulence of the intracellular and extracellular fluid mediums at high velocities.

Bodor [44], however, proposed that MMG amplitude may be more related to power output than PT during maximal concentric isokinetic muscle actions. Cramer et al. [45] tested this hypothesis by examining the potential relationship between MMG amplitude and mean power output during maximal concentric isokinetic leg extensions at velocities ranging from 60 to 300°·s^-1^. The authors [45] reported that leg extension PT decreased with increases in velocity, but mean power output and MMG amplitude for the rectus femoris, vastus lateralis and vastus medialis muscles increased from 60 to 240°·s^-1^, and then plateaued from 240 to 300°·s^-1^. It was suggested that MMG amplitude may be useful for monitoring training-induced changes in power output.

Interestingly, there is also evidence to suggest that there may be muscle-specific differences in the MMG amplitude responses with increases in velocity during maximal concentric isokinetic muscle actions. For example, Cramer et al. [46] reported that during maximal concentric isokinetic leg extensions at velocities ranging from 60 to 300°·s^-1^, MMG amplitude for each of the superficial quadriceps femoris muscles (rectus femoris, vastus lateralis, and vastus medialis) increased with velocity from 60 to 180°·s^-1^. At velocities above 180°·s^-1^, however, MMG amplitude increased to 240°·s^-1 ^and then plateaued from 240 to 300°·s^-1 ^for the vastus lateralis, plateaued from 180 to 300°·s^-1 ^for the rectus femoris, and increased from 180 to 300°·s^-1 ^for the vastus medialis. It was suggested that the muscle-specific differences in the MMG amplitude responses may be due to differences among the rectus femoris, vastus lateralis, and vastus medialis muscles in fiber type composition and/or muscle architecture (unipennate versus bipennate or the degree of pennation). In addition, Ebersole et al. [47] examined the patterns for MMG amplitude from the vastus lateralis with increases in velocity during maximal concentric isokinetic and passive leg extension muscle actions. The authors [47] found that MMG amplitude increased with leg extension velocity during both the active and passive leg extension muscle actions. During the passive leg extension muscle actions, however, the vastus lateralis muscle remained inactive because the EMG amplitude values from the muscle were very small and did not change with increases in velocity. It was hypothesized that the velocity-related increases in MMG amplitude for the vastus lateralis muscle may have been due to greater turbulence of the intracellular and extracellular fluid mediums and/or cross-talk from the hamstring muscles. Thus, these findings suggested that in addition to power output, MMG amplitude may be affected by factors such as fiber type composition and muscle architecture [46], as well as turbulence of the intracellular and extracellular fluid mediums and/or cross-talk [47]. Cramer et al. [48], however, recently reported that the potential for cross-talk in surface MMG signals is relatively small, even for muscles that are very close to each other and have a common innervation. Specifically, the authors [48] used cross-correlation to quantify the common variance present in the MMG signals from the rectus femoris, vastus lateralis, and vastus medialis muscles during maximal concentric and eccentric isokinetic leg extensions at a velocity of 60°·s^-1^. The common variance shared between the MMG signals from any two muscles ranged from 14% to 27%, and it was suggested [48] that, "...despite the potential for some cross-talk, MMG measurements can be used to examine differences between the patterns of MMG amplitude and frequency responses of the superficial muscles of the quadriceps femoris."

One issue that is still unresolved, however, is the potential relationship between MMG MPF and velocity during maximal concentric isokinetic muscle actions. For example, Cramer et al. [49] reported that during maximal concentric isokinetic leg extensions, MMG MPF for the rectus femoris, vastus lateralis, and vastus medialis muscles did not change with increases in velocity from 60 to 240°·s^-1^. There were, however, velocity-related increases in MMG MPF (marginal means, collapsed across muscles) from 240 to 300°·s^-1^. Furthermore, Cramer et al. [50] recently suggested that the MMG MPF responses to increases in velocity may be muscle-specific. For example, during maximal concentric isokinetic leg extensions, there were velocity-related increases in MMG MPF for the rectus femoris and vastus lateralis muscles, but not for the vastus medialis [50]. Thus, it is possible that potential increases in MMG MPF during concentric isokinetic muscle actions may be muscle- and/or velocity-specific.

**Figure 2:**
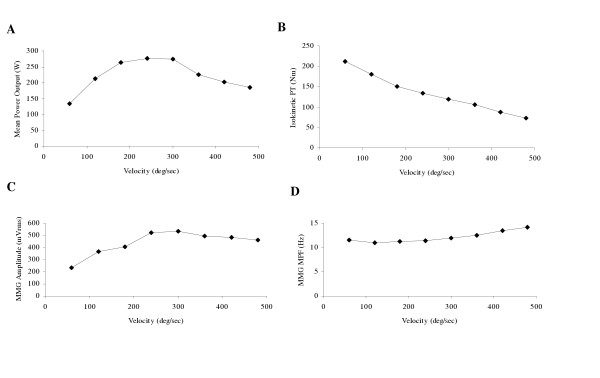
**Mean power output, isokinetic peak torque (PT), mechanomyographic (MMG) amplitude, and MMG mean power frequency (MPF) responses with increases in velocity during maximal concentric isokinetic muscle actions**. **(A) **Relationship between mean power output and angular velocity during maximal concentric isokinetic leg extensions. **(B) **Relationship between maximal concentric isokinetic leg extension peak torque and angular velocity. **(C) **Relationship between mechanomyographic (MMG) amplitude (averaged across the rectus femoris, vastus lateralis, and vastus medialis muscles) and angular velocity. **(D) **Relationship between MMG mean power frequency (MPF, averaged across the rectus femoris, vastus lateralis, and vastus medialis muscles) and angular velocity. The data presented in each graph are the mean values from 26 subjects, and all MMG signals were recorded with a piezoelectric contact sensor (HP 21050A).

Collectively, the results from these studies have indicated that during maximal concentric isokinetic muscle actions, there are velocity-related increases in MMG amplitude that may be due to reduced muscle stiffness, a greater rate of cross-bridge cycling, and/or increased turbulence of the intracellular and extracellular fluid mediums. In addition, MMG amplitude may be closely related to power output (see Figure 2), which suggests that MMG could potentially be useful for examining training-induced adaptations in muscular power. It is unclear, however, if there is a relationship between MMG MPF and velocity during concentric isokinetic muscle actions.

## MMG amplitude and frequency responses with increases in velocity during maximal eccentric isokinetic muscle actions

Eccentric muscle actions may require unique motor control strategies from the central nervous system [51]. Specifically, it has been suggested that when compared to concentric and/or isometric activities, eccentric muscle actions are characterized by lower levels of muscle activation [26], reduced recruitment thresholds [52,53], lower motor unit firing rates [54], de-recruitment of low threshold slow-twitch motor units [55], and selective activation of high threshold fast-twitch motor units [56]. In addition, unlike maximal concentric isokinetic muscle actions, torque production during maximal eccentric isokinetic muscle actions does not change with increases in velocity [37,38,57-59], but there are significant velocity-related increases in mean power output [59]. Thus, MMG amplitude and/or MPF could provide information regarding the unique motor control strategies that are used during eccentric muscle actions. In addition, the possibility for velocity-related increases in mean power output suggests that maximal eccentric isokinetic muscle actions may be useful for testing the hypothesis of Bodor [44] that MMG amplitude is more closely related to power output than PT during maximal isokinetic muscle actions.

Smith et al. [57] examined the MMG amplitude responses from the vastus lateralis during maximal eccentric isokinetic muscle actions of the leg extensors at velocities of 60, 90, 120, and 180°·s^-1^. The authors [57] found that although there was no change in eccentric PT from 60 to 180°·s^-1^, there was a velocity-related increase in MMG amplitude for the vastus lateralis muscle. In addition, the MMG amplitude values during the maximal eccentric isokinetic muscle actions were highly reliable, with ICCs ranging from R = 0.97–0.98 and no significant differences between the mean MMG amplitude values for test versus retest at any velocity. The authors [57] proposed three potential hypotheses to explain the velocity-related increase in MMG amplitude for the vastus lateralis muscle. First, an increased rate of cross bridge activity during high-velocity eccentric muscle actions could result in larger vibrations of the myosin heads, potentially resulting in greater MMG amplitude values. Second, de-recruitment of low threshold slow-twitch motor units [55] and selective activation of high threshold fast-twitch motor units [56] at high velocities during eccentric muscle actions could influence MMG amplitude because in some muscles, fast-twitch motor units may be more superficially located than slow-twitch motor units [60]. Thus, when compared to slow-twitch motor units, the vibrations from fast-twitch motor units may be damped to a lesser degree by the surrounding tissues, potentially resulting in greater MMG amplitude values [57]. Finally, the velocity-related increases in MMG amplitude may be due to faster movement of the limb and a greater overall disturbance of the intracellular and extracellular fluid mediums [57].

Evetovich et al. [38] also reported velocity-related increases in MMG amplitude for the vastus lateralis during maximal eccentric isokinetic muscle actions of the leg extensors. There was no gender difference for the increases in MMG amplitude with velocity, but the men demonstrated greater MMG amplitude values than the women at all velocities. As stated previously for maximal concentric isokinetic muscle actions, the greater MMG amplitude values for men during maximal eccentric isokinetic muscle actions could be related to a larger muscle mass and/or thinner adipose tissue layer over the vastus lateralis than is typically found in women [38]. In addition, Smith et al. [37] reported that MMG amplitude for the biceps brachii increased with velocity during maximal eccentric isokinetic muscle actions of the forearm flexors at velocities of 30, 90, and 150°·s^-1^. These findings indicated that increases in MMG amplitude with velocity during maximal eccentric isokinetic muscle actions can occur in the biceps brachii, which is a fusiform muscle with a potentially different fiber type composition [60] when compared to the unipennate vastus lateralis that had been examined in previous studies [38,57]. The exact mechanism(s) underlying the velocity-related increase in MMG amplitude was, however, still unknown.

Theoretically, de-recruitment of low threshold slow-twitch motor units [55] and selective activation of high threshold fast-twitch motor units [56] during high-velocity eccentric muscle actions could result in greater MMG amplitude and MPF values. Evetovich et al. [61], however, reported that during maximal eccentric isokinetic muscle actions of the leg extensors at velocities of 60, 120, and 180°·s^-1^, there was a velocity-related increase in MMG amplitude for the vastus lateralis muscle, but there was no change in MMG MPF from 60 to 180°·s^-1^. It was suggested that the increase in MMG amplitude may have been due to factors other than a velocity-related shift in the contributions of slow- and fast-twitch muscle fibers to torque production. In addition, Cramer et al. [62] found that during maximal eccentric isokinetic muscle actions of the leg extensors, MMG MPF for the rectus femoris, vastus lateralis, and vastus medialis muscles actually decreased with an increase in velocity from 60 to 120°·s^-1^, and then remained relatively stable from 120 to 180°·s^-1^. Although there was no change in eccentric PT, there were increases in mean power output and the average MMG amplitude values for each muscle from 60 to 180°·s^-1^. Furthermore, Cramer et al. [59] reported that mean power output and MMG amplitude for the vastus lateralis increased with velocity during maximal eccentric isokinetic muscle actions of the leg extensors at velocities of 30, 90, and 150°·s^-1^. The velocity-related increases in MMG amplitude were similar, however, in men and women.

**Figure 3:**
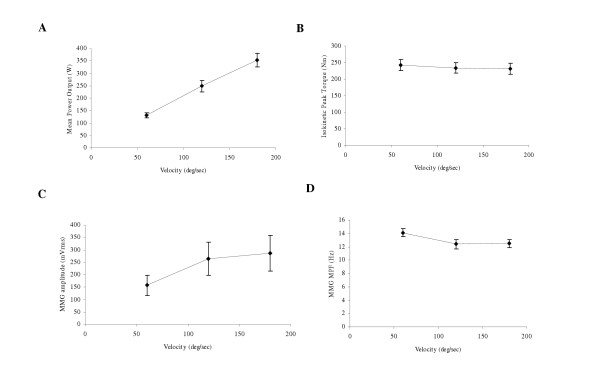
**Mean power output, isokinetic peak torque (PT), mechanomyographic (MMG) amplitude, and MMG mean power frequency (MPF) responses with increases in velocity during maximal eccentric isokinetic muscle actions**. **(A) **Relationship between mean power output and angular velocity during maximal eccentric isokinetic muscle actions of the leg extensors. **(B) **Relationship between isokinetic peak torque and angular velocity during maximal eccentric isokinetic muscle actions of the leg extensors. **(C) **Relationship between mechanomyographic (MMG) amplitude (averaged across the rectus femoris, vastus lateralis, and vastus medialis muscles) and angular velocity. **(D) **Relationship between MMG mean power frequency (MPF, averaged across the rectus femoris, vastus lateralis, and vastus medialis muscles) and angular velocity. The data presented in each graph are the mean values from 24 subjects, and all MMG signals were recorded with a piezoelectric contact sensor (HP 21050A).

Thus, the results from these studies [37,38,57,59,61,62] provided support for the hypothesis that MMG amplitude could be more closely related to power output than PT during maximal isokinetic (both concentric and eccentric) muscle actions (see Figure 3) [44]. In addition, the velocity-related increases in MMG amplitude that have been reported in the biceps brachii [37], vastus lateralis [38,57], rectus femoris, and vastus medialis muscles [59,62] suggested that MMG amplitude increases with velocity during maximal eccentric isokinetic muscle actions, even for muscles with a potentially different architecture (fusiform, unipennate, bipennate) and/or fiber type composition. Finally, MMG MPF may not reflect a potential velocity-related shift in the contributions of fast- and slow-twitch muscle fibers to torque production during maximal eccentric isokinetic muscle actions.

## MMG amplitude and frequency responses with increases in torque during incremental concentric and eccentric muscle actions

Dalton and Stokes [26,63] were the first investigators to examine the potential torque-related patterns for MMG amplitude and MPF during concentric and eccentric muscle actions. The authors [26,63] measured the MMG signal from the biceps brachii muscle during submaximal dynamic constant external resistance (DCER, formerly called isotonic) muscle actions of the forearm flexors in which the subjects lifted (concentric) and lowered (eccentric) weights ranging from 0 to 8.5 kg. Dalton and Stokes [26] reported that MMG amplitude for the biceps brachii increased linearly from 0 to 8.5 kg during both the concentric and eccentric muscle actions. In addition, during the concentric muscle actions, MMG MPF for the biceps brachii increased from 0 to approximately 5.5 kg and then decreased from 5.5 to 8.5 kg [63]. During the eccentric muscle actions, however, MMG MPF remained relatively stable from 0 to 8.5 kg [63]. It was suggested that during submaximal concentric muscle actions of the forearm flexors, torque may be increased by recruiting additional motor units in the biceps brachii, as well as increasing their firing rates. During submaximal eccentric muscle actions, however, forearm flexion torque may be increased primarily through motor unit recruitment, with little change in motor unit firing rates. Petitjean et al. [64] also examined the potential relationship between MMG amplitude and torque during submaximal concentric DCER muscle actions of the forearm flexors. The authors [64] reported that MMG amplitude for the biceps brachii and brachioradialis muscles increased linearly with forearm flexion torque. Beck et al. [27,65] reported similar results for the biceps brachii during submaximal to maximal concentric isokinetic muscle actions of the forearm flexors at a velocity of 30°·s^-1^. Specifically, MMG amplitude for the biceps brachii muscle increased linearly with forearm flexion torque, but there was no significant change in MMG MPF from 10% to 100% PT. The findings for MMG MPF differed from those of Dalton and Stokes [63]. It is possible, however, that the discrepancies among the results from these studies [27,63,65] may have been due to differences in experimental design. Specifically, Dalton and Stokes [63] used submaximal DCER muscle actions of the forearm flexors with weights ranging from 0 to 8.5 kg, while the subjects in the studies by Beck et al. [27,65] performed maximal as well as submaximal isokinetic muscle actions at a single velocity (30°·s^-1^) over a range of 10% to 100% PT. Beck et al. [27,65] suggested, however, that the torque-related increases in MMG amplitude for the biceps brachii, with no significant change in MMG MPF, may have been due to recruitment, with little change in motor unit firing rates. In addition, Coburn et al. [66] examined the MMG amplitude and MPF versus torque relationships for the vastus medialis during submaximal to maximal concentric isokinetic muscle actions of the leg extensors at a velocity of 30°·s^-1^. The authors [66] reported that MMG amplitude and MPF for the vastus medialis muscle increased linearly with leg extension torque from 10% to 100% PT. It was suggested that the torque-related increases in MMG amplitude and MPF for the vastus medialis may have been due to concurrent modulation of the number of active motor units and their firing rates throughout the entire range of concentric leg extension torque.

Recent studies [28,67] have also examined the MMG amplitude and MPF versus eccentric torque relationships. Beck et al. [67] found that during submaximal to maximal eccentric isokinetic muscle actions of the forearm flexors at a velocity of 30°·s^-1^, MMG amplitude for the biceps brachii increased from 10% to approximately 60% PT and then plateaued from 60% to 100% PT. In addition, there was a linear increase in MMG MPF for the biceps brachii from 10% to 100% PT. It was hypothesized that the increases in MMG amplitude and MPF from 10% to approximately 60% PT were likely due to recruitment of motor units in the biceps brachii muscle, as well as increases in their firing rates. The plateau in MMG amplitude, and increase in MMG MPF from 60% to 100% PT, however, suggested that recruitment in the biceps brachii muscle may have ended at approximately 60% PT, and further increases in eccentric isokinetic torque were due to changes in motor unit firing rates [67]. In addition, Coburn et al. [28] examined the MMG amplitude and MPF versus torque relationships for the vastus medialis during submaximal to maximal eccentric isokinetic muscle actions of the leg extensors at a velocity of 30°·s^-1^. The authors [28] reported that both MMG amplitude and MPF for the vastus medialis muscle increased linearly with eccentric isokinetic torque from 10% to 100% PT. It was suggested that for the vastus medialis muscle, motor unit recruitment and firing rate modulation may occur throughout the entire range of eccentric torque production. Interestingly, Madeleine et al. [68] found that during both concentric and eccentric muscle actions of the first dorsal interosseous, there were no changes in MMG amplitude or MPF with increases in torque. These findings are in contrast to the torque-related increases in MMG amplitude and/or MPF that have been reported for the biceps brachii [26,27,63-65,67] and vastus medialis [28,66,69] during concentric and eccentric muscle actions, and may reflect muscle-specific differences in the motor control strategies that modulate torque production. For example, previous studies [14,33,70] have reported that large limb muscles such as the biceps brachii and vastus medialis rely heavily on motor unit recruitment for increasing isometric torque production. For smaller hand muscles such as the first dorsal interosseous, however, increasing motor unit firing rates is important for generating additional torque, particularly above approximately 50% MVC when all motor units have been recruited [14,33]. Thus, if these muscle-specific differences in motor control strategies also occur during concentric and eccentric muscle actions, then the lack of significant changes in MMG amplitude and MMG MPF reported by Madeleine et al. [68] could be related to the potential importance of firing rate modulation for increasing concentric and eccentric torque production in the first dorsal interosseous muscle.

**Figure 4:**
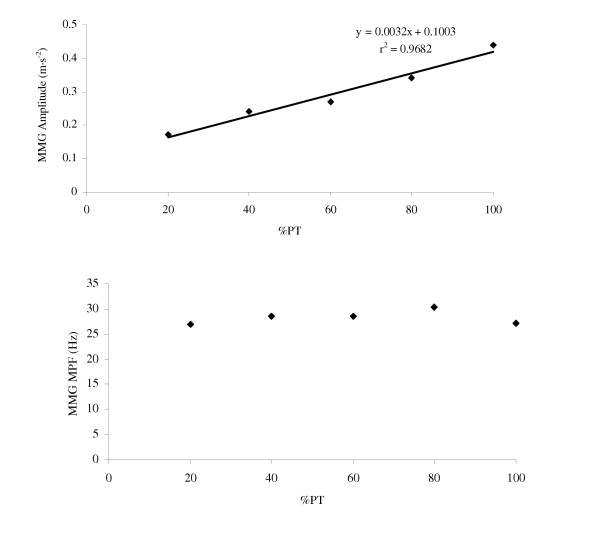
**Relationships for mechanomyographic (MMG) amplitude and mean power frequency (MPF) versus torque during concentric isokinetic muscle actions**. The *top graph *shows the linear relationship between mechanomyographic (MMG) amplitude (m·s^-2^) for the biceps brachii and concentric isokinetic torque. The *bottom graph *demonstrates the lack of a significant relationship between MMG mean power frequency (MPF, Hz) for the biceps brachii and concentric isokinetic torque. The data presented are from one subject, and the MMG signals were recorded with an accelerometer (Entran EGAS FT 10) during concentric isokinetic forearm flexion muscle actions at a velocity of 30°·s^-1^.

**Figure 5:**
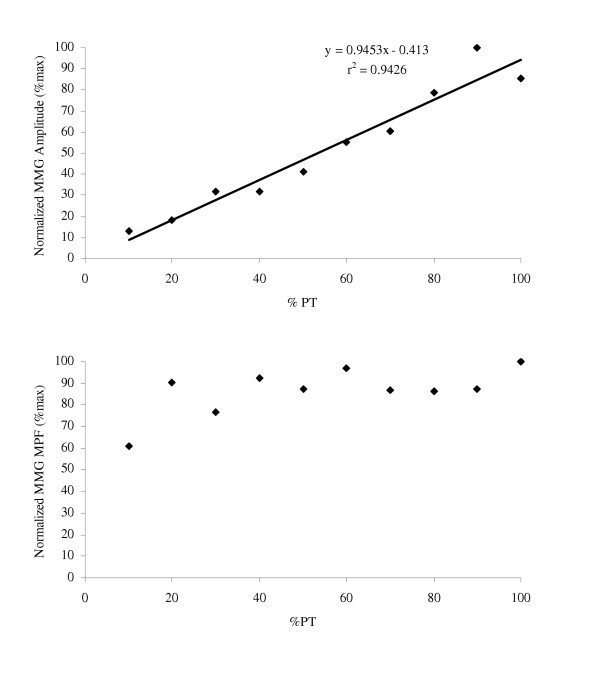
**Relationships for mechanomyographic (MMG) amplitude and mean power frequency (MPF) versus torque during eccentric isokinetic muscle actions**. The *top graph *shows the linear relationship between normalized mechanomyographic (MMG) amplitude and percent eccentric isokinetic peak torque (PT) for the vastus medialis muscle of one subject. The *bottom graph *demonstrates that for this particular subject (the same subject as in the *top graph*), there was no relationship between normalized MMG mean power frequency (MPF) for the vastus medialis muscle and percent eccentric isokinetic PT. The data presented are from one subject, and the MMG signals were recorded with a piezoelectric contact sensor during eccentric isokinetic muscle actions of the leg extensors at a velocity of 30°·s^-1^.

Collectively, the results from these studies [26-28,63-69] suggested that the torque-related patterns for MMG amplitude and MPF may provide information regarding the motor control strategies that modulate torque in various muscles during concentric and eccentric muscle actions (see Figures 4 and 5). In addition, the MMG amplitude and/or MPF versus dynamic torque relationships may be useful in rehabilitative settings, where functional activities such as dynamic muscle actions are usually preferred over isometric muscle actions [6]. In particular, the linear MMG amplitude versus torque relationship during concentric muscle actions of the biceps brachii [26,27,64] and vastus medialis [66] suggested that MMG amplitude could potentially be used to assess dynamic force production in situations where force cannot be measured directly (i.e. when examining some of the individual limb muscles, the facial muscles, and/or the muscles of the back and abdomen) [23].

## MMG amplitude and MPF responses during fatiguing concentric and eccentric isokinetic muscle actions

Recent studies have also investigated the MMG amplitude and/or MPF responses during fatiguing concentric [71,72] and eccentric [73] isokinetic muscle actions. For example, Perry-Rana et al. [71] examined the patterns for MMG amplitude from the rectus femoris, vastus lateralis, and vastus medialis muscles during 50 consecutive maximal concentric isokinetic leg extensions at velocities of 60, 180, and 300°·s^-1^. The authors [71] found that at 60 and 300°·s^-1^, there were quadratic decreases in MMG amplitude for the vastus lateralis and vastus medialis muscles, but linear decreases in MMG amplitude for the rectus femoris. At 180°·s^-1^, MMG amplitude decreased quadratically for the vastus medialis muscle, but there were linear decreases in MMG amplitude for the rectus femoris and vastus lateralis [71]. The reductions in MMG amplitude at each velocity (60, 180, and 300°·s^-1^) were greater for the rectus femoris muscle than for the vastus lateralis and vastus medialis. In addition, at 60°·s^-1^, the decrease in concentric isokinetic leg extension torque was best fit with a quadratic model, but at 180 and 300°·s^-1^, the patterns for torque were best fit with cubic models [71]. It was suggested that the decreases in MMG amplitude for each muscle at the three velocities may have been due to reduced muscle compliance and/or "muscle wisdom," in which the central nervous system reduces motor unit firing rates to compensate for fatigue-induced increases in muscle fiber relaxation times [74]. Theoretically, muscle wisdom allows for an economical activation of fatiguing muscle [75] and could reduce the number of pressure waves recorded per unit of time, thereby decreasing MMG amplitude [71,76]. In addition, it has been suggested [77-80] that during prolonged static and dynamic muscle actions, there are increases in muscle thickness, fluid content, and intramuscular pressure, all of which may contribute to reductions in the compliance (i.e. elastic properties) of the muscle. In theory, a decrease in muscle compliance could restrict the muscle fiber oscillations and/or attenuate the pressure waves that generate the MMG signal, thereby decreasing MMG amplitude [71]. Søgaard et al. [81], however, recently suggested that MMG amplitude may not be influenced by changes in intramuscular pressure. Specifically, the authors [81] reported that during an isometric ramp muscle action of the forearm flexors from 10% to 60% MVC, MMG amplitude for the biceps brachii increased linearly with torque, even when intramuscular pressure was increased with a sphygmomanometer cuff. It was suggested that MMG amplitude may not be influenced by intramuscular pressure. This hypothesis should be tested, however, at higher torque levels and with different muscles to further examine the potential influence of intramuscular pressure on MMG amplitude. Furthermore, the greater decreases in MMG amplitude for the rectus femoris muscle than for the vastus lateralis and vastus medialis reported by Perry-Rana et al. [71] may have reflected a larger percentage of fast-twitch muscle fibers in the rectus femoris [60]. Beck et al. [72] examined the MMG amplitude and MPF responses from the biceps brachii during 50 consecutive maximal concentric isokinetic muscle actions of the forearm flexors at a velocity of 180°·s^-1^. The authors [72] found linear decreases in both MMG amplitude and MPF across the 50 repetitions. In addition, the decrease (approximately 70%) in mean forearm flexion torque was best fit with a cubic model [72]. It was suggested that the reductions in MMG amplitude and MPF may have been due to fatigue-induced decreases in motor unit firing rates (i.e. muscle wisdom) and/or reduced compliance in the biceps brachii muscle. Beck et al. [72] also hypothesized, however, that de-recruitment of fast-twitch motor units [82,83], which have higher firing rates than slow-twitch motor units [40,41,84], could, theoretically, result in decreases in both MMG amplitude and MPF. In addition, Perry-Rana et al. [73] examined the MMG amplitude responses from the rectus femoris, vastus lateralis, and vastus medialis during 25 consecutive maximal eccentric isokinetic muscle actions of the leg extensors at a velocity of 120°·s^-1^. The authors [73] found that for the vastus lateralis and vastus medialis muscles, MMG amplitude decreased linearly across the 25 repetitions. For the rectus femoris muscle, however, the MMG amplitude pattern was best fit with a cubic model, in which MMG amplitude increased slightly during repetitions 1–5, decreased during repetitions 5–20, and increased throughout the last 5 repetitions of the test. Interestingly, the pattern for eccentric leg extension torque was best fit with a cubic model, where torque increased during the first 10 repetitions, and then remained relatively stable from repetitions 11–25. The authors [73] hypothesized that the decreases in MMG amplitude for each muscle may have been due to the effects of muscle wisdom and/or reduced muscle compliance. In addition, it was suggested that the muscle-specific differences in the MMG amplitude patterns (i.e. a cubic decline for the rectus femoris versus linear decreases for the vastus lateralis and vastus medialis) may have been due to differences among the three muscles in fiber type composition and/or architecture (unipennate versus bipennate).

Thus, the results from these studies [71-73] suggested that MMG amplitude and/or MPF could potentially provide information regarding the motor control strategies that are used during fatiguing concentric and eccentric isokinetic muscle actions. Specifically, fatigue-induced decreases in MMG amplitude and MPF may reflect reductions in motor unit firing rates (muscle wisdom) and/or de-recruitment of fast-twitch motor units [71-74,82]. In addition, MMG amplitude and/or MPF patterns during fatiguing isokinetic muscle actions may be influenced by fiber type composition and/or muscle architecture [71,73]. Finally, decreases in muscle compliance during repeated maximal isokinetic muscle actions [77-80] could attenuate the muscle fiber oscillations and/or pressure waves that generate the MMG signal.

## MMG amplitude and MPF responses during cycle ergometry

Several studies [29-32,85-87] have investigated the MMG amplitude and/or MPF responses during cycle ergometry. For example, Stout et al. [29] examined the patterns for oxygen consumption rate (VO_2_) and MMG amplitude from the vastus lateralis muscle during an incremental cycle ergometer test performed to exhaustion. The authors [29] found that the MMG amplitude and VO_2_ versus power output relationships were highly linear (r^2 ^range = 0.79–0.99 and 0.97–0.99, respectively). In addition, for 20 of the 24 subjects that participated in the study, the linear slope coefficients for the normalized MMG amplitude and VO_2_ versus power output relationships were statistically equivalent. It was suggested that MMG could be useful for quantifying muscular activity and monitoring changes in exercise intensity during incremental cycle ergometry. Furthermore, the similar slope coefficients for the normalized MMG amplitude and VO_2_ versus power output relationships for the majority of the subjects indicated that there may be a close relationship between the metabolic (VO_2_) and mechanical (MMG) aspects of muscle activity during cycle ergometry. Shinohara et al. [30] also found that MMG amplitude for the vastus lateralis muscle increased linearly with power output during incremental cycle ergometry. In fact, MMG amplitude was more linearly related to power output than was EMG amplitude, which tended to increase curvilinearly at high power outputs. Perry et al. [31] reported similar results for the vastus lateralis muscle during incremental cycle ergometry. Specifically, for the majority of the subjects (7 out of 9), MMG amplitude increased linearly with power output, but EMG amplitude increased curvilinearly. The authors [31] also reported linear increases in normalized heart rate and ratings of perceived exertion (RPE) with power output, and the linear slope coefficients for these relationships were statistically equivalent to the slope coefficient for the linear normalized MMG amplitude versus power output relationship. Thus, it was suggested that during incremental cycle ergometry, there may be a close relationship among the mechanical (MMG), heart rate, and perception of effort (RPE) aspects of muscular activity.

The potential relationship between MMG frequency and motor unit firing rates [5,13,84] has suggested that the frequency domain of the MMG signal may provide information regarding the motor control strategies that are used during incremental cycle ergometry [32]. Theoretically, increases in MMG MPF could reflect modulation of motor unit firing rates with increasing power output. Perry et al. [32], however, reported no change in MMG MPF for the vastus lateralis muscle, but a linear increase in MMG amplitude with increases in power output during an incremental cycle ergometer test performed to exhaustion. It was suggested that during cycle ergometry, motor control strategies are examined across a narrow range of very low levels of force production. Specifically, Sjøgaard [88] reported that during cycle ergometry at 100% of VO_2_ max and a pedal cadence of 60 rev·min^-1^, the force exerted against the pedals represented approximately 16% of the isometric MVC. Large limb muscles (such as the vastus lateralis) typically rely heavily on recruitment for increasing isometric torque to about 50–80% MVC, above which, modulation of motor unit firing rates becomes progressively more important for increasing torque [33,70]. Thus, the lack of a significant change in MMG MPF for the vastus lateralis reported by Perry et al. [32] may have been due to the low force levels examined (relative to maximal capabilities) and recruitment as the primary motor control strategy for increasing power output, rather than firing rate modulation.

Several studies [85-87] have also examined the MMG amplitude responses during continuous cycle ergometry performed at constant, submaximal power outputs. For example, Housh et al. [87] investigated the patterns for MMG amplitude for the vastus lateralis and vastus medialis muscles during continuous, constant power output workbouts at 50, 65, 80, and 95% of the peak power (Wpeak) that was achieved during an incremental cycle ergometer test performed to exhaustion. The authors [87] found that the MMG amplitude responses were dependent upon the power output at which the workbout was performed, as well as the muscle that was being examined. Specifically, MMG amplitude for the vastus lateralis and vastus medialis muscles decreased during the workbouts at 50% and 65% Wpeak, but remained stable at 80% Wpeak. At 95% Wpeak, however, MMG amplitude increased for the vastus medialis muscle, but remained relatively stable for the vastus lateralis. It was suggested that the decreases in MMG amplitude for both muscles (vastus lateralis and vastus medialis) at 50% and 65% Wpeak may have been due to fatigue-induced decreases in motor unit firing rates (i.e. muscle wisdom). At 80% Wpeak, however, the lack of a significant change in MMG amplitude for either the vastus lateralis or vastus medialis may have reflected a balance between the influences of recruitment (which can increase MMG amplitude) and decreases in motor unit firing rates (which can reduce MMG amplitude). In contrast, the increases in MMG amplitude for the vastus medialis muscle at 95% Wpeak suggested that recruitment may have had a greater influence on MMG amplitude than potential fatigue-induced decreases in motor unit firing rates. The vastus lateralis muscle, however, demonstrated no change in MMG amplitude at 95% Wpeak, and it was hypothesized that the tendonous iliotibial band that covers the vastus lateralis may have affected the muscle fiber oscillations that were being transmitted to the skin surface, thereby influencing MMG amplitude [87]. Perry et al. [85] also examined the MMG amplitude responses from the vastus lateralis muscle during continuous, constant power output cycle ergometry. The authors [85] found that MMG amplitude decreased during continuous workbouts at 28%, 35%, and 42% Wpeak. These findings were consistent with those of Housh et al. [87] for the vastus lateralis and vastus medialis muscles at 50% and 65% Wpeak, and it was hypothesized that the decreases in MMG amplitude for the vastus lateralis may have been due to the effects of muscle wisdom, and/or decreases in muscle compliance. In addition, Bull et al. [86] examined the MMG amplitude responses from the vastus lateralis muscle during continuous cycle ergometry at a submaximal workload known as critical power (CP). In theory, CP is the maximal power output that can be accomplished without fatigue [89,90] and, therefore, should be characterized by steady state VO_2_ and no increase in muscle activation [86]. Thus, MMG amplitude could also remain stable at CP, potentially reflecting little change in the motor unit mechanical activities that are required to perform the workload. Bull et al. [86], however, found that there was a quadratic decrease in MMG amplitude for the vastus lateralis muscle during a 60-min cycle ergometer workbout at CP. It should be noted that different mathematical models have been used to estimate CP [91], and Housh et al. [92] found that CP was approximately 17% greater than the power output that could be maintained for 60-min. Thus, in some cases, CP may be a fatiguing workload, and it is possible that fatigue-related factors such as muscle wisdom and/or reduced muscle compliance may have contributed to the decreases in MMG amplitude reported by Bull et al. [86] for the vastus lateralis muscle.

**Figure 6:**
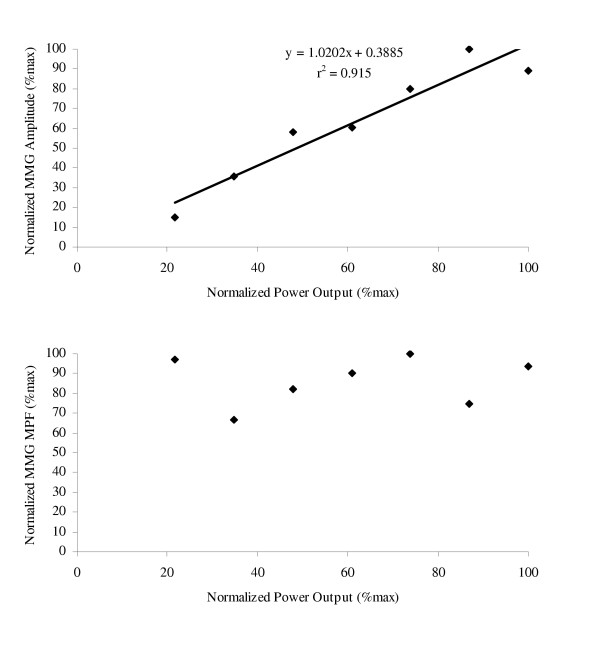
**Relationships for mechanomyographic (MMG) amplitude and mean power frequency (MPF) versus power output during incremental cycle ergometry**. The *top graph *shows the linear relationship between normalized mechanomyographic (MMG) amplitude (%max) for the vastus lateralis muscle and normalized power output. The *bottom graph *demonstrates the lack of a significant relationship between normalized MMG mean power frequency (MPF, %max) for the vastus lateralis muscle and normalized power output. The data presented are from one subject, and the MMG signals were recorded with a contact sensor (HP 21050A) during an incremental cycle ergometer test to exhaustion.

Collectively, the results from these studies provided several important pieces of information regarding the MMG amplitude and frequency responses during cycle ergometry. Specifically, the highly linear relationship between MMG amplitude and power output during incremental cycle ergometry (see Figure 6) [29-32] provided indirect support for the hypothesis of Bodor [44] that during maximal isokinetic muscle actions, MMG amplitude may be more closely related to power output than force production. In addition, the lack of a significant change in MMG MPF for the vastus lateralis muscle reported by Perry et al. [32] suggested that during incremental cycle ergometry, firing rate modulation may not be as important as recruitment for increasing power output. Furthermore, the MMG amplitude responses during continuous, constant power output cycle ergometry are dependent on the relative workload that is being performed and may be related to the competing influences of fatigue-related decreases in motor unit firing rates and increases in motor unit recruitment. In particular, decreases in MMG amplitude for workloads below approximately 65% Wpeak may be due to fatigue-induced decreases in motor unit firing rates (muscle wisdom) and/or reduced muscle compliance, while an increase in MMG amplitude at 95% Wpeak may reflect a greater contribution of motor unit recruitment (which can increase MMG amplitude) than decreases in firing rates (which can decrease MMG amplitude) to the MMG signal [85-87]. Finally, the relatively stable MMG amplitude values during continuous cycle ergometry at 80% Wpeak may have been due to approximately equal influences of motor unit recruitment and decreases in firing rates on the MMG signal [87].

## Effects of dynamic resistance training on MMG amplitude and frequency

Several studies [93-97] have investigated the potential for MMG to be used as a tool for examining the neuromuscular adaptations that occur during a resistance training program. Resistance training results in increases in muscle fiber size (hypertrophy) [98,99] and muscle stiffness [100], and it has been suggested [94,95] that both of these factors may influence the amplitude and/or frequency contents of the MMG signal. For example, Evetovich et al. [94] hypothesized that hypertrophy could decrease the distance between the muscle and the MMG sensor as the fibers press against the tissue layers superficial to the muscle (i.e. fascia, adipose tissue, etc.). The tissue between the muscle and the MMG sensor acts as a low pass filter for the muscle fiber oscillations and pressure waves that generate the MMG signal [4,25]. Thus, the close proximity of hypertrophied muscle fibers to the MMG sensor may result in less attenuation of the MMG signal by the tissue layers superficial to the muscle, resulting in greater MMG amplitude values [94]. In addition, it has been suggested [95] that training-induced increases in muscle stiffness could affect MMG frequency. Specifically, Barry and Cole [101] indicated that the MMG signal occurs at the "...resonant frequency of the muscle," which is a function of the mass, length, topology, and stiffness of the muscle being investigated. It was suggested, however, that during an isometric muscle action, the changes in stiffness are greater than the changes in any of the other parameters [101]. In addition, both the resonant frequency and peak frequency of the MMG signal increased with force production during an electrically-stimulated tetanic contraction of isolated frog gastrocnemius muscle [101]. Thus, these findings suggested that training-induced increases in muscle stiffness could potentially result in corresponding increases in the muscle's resonant frequency (and, by extension, MMG frequency).

Cerquiglini et al. [93] examined the effects of two months of DCER strength training on MMG frequency in two sedentary subjects and two Olympic weightlifters. During the training, the subjects performed the press, jerk, and snatch exercises with loads corresponding to 80% of their personal records and were tested weekly for "...maximum isometric strength of the lower limbs..." in two "typical lifting positions" [93]. The MMG signals were recorded from the vastus lateralis muscle and the medial head of the gastrocnemius during the isometric strength tests. The authors [93] found that the resistance training program resulted in a "...relative increase of higher frequencies (above 70 Hz)" in the MMG signals from the vastus lateralis and gastrocnemius muscles. It was suggested [93] that the information provided by the MMG signal was "...meaningful as regards the effect of training on muscle..." and could potentially be used by trainers and/or athletes to monitor changes in muscle function during a resistance training program. Evetovich et al. [94,95] examined the effects of concentric isokinetic strength training of the leg extensors on MMG amplitude and MPF for the vastus lateralis muscle. Twenty-one male subjects that were not experienced in resistance training were randomly assigned into either a training or control group. The subjects in the training group performed maximal concentric isokinetic leg extensions with the non-dominant limb at a velocity of 90°·s^-1 ^three times per week for twelve weeks. The subjects in the training and control groups were tested for maximal concentric isokinetic leg extension PT at a velocity of 90°·s^-1 ^prior to beginning the twelve week training program (week 0), and every four weeks thereafter (weeks 4, 8, and 12, respectively). During each testing session, the MMG signal was recorded from the vastus lateralis muscle. The authors [94,95] found that there was a significant increase in leg extension PT across the twelve week training period for the training group, but not for the control group. There were, however, no changes in MMG amplitude or MPF for the vastus lateralis muscle from week 0 to week 12 for either group. It was suggested that the lack of a significant change in MMG amplitude and MPF following training may have been due to compression of hypertrophied muscle fibers in the vastus lateralis by the tendonous iliotibial band and/or training-induced adaptations in muscles other than the vastus lateralis. Specifically, although muscle hypertrophy was not directly examined [94,95], increases in muscle fiber size could potentially stretch the iliotibial band, resulting in a taut layer of fascia that compresses the vastus lateralis and restricts the muscle fiber oscillations that generate the MMG signal. Theoretically, this could influence MMG amplitude and/or MPF. It is also possible, however, that training-induced adaptations in muscles other than the vastus lateralis may have contributed to increases in leg extension PT, with no changes in MMG amplitude and MPF. For example, reduced hamstring coactivation [102] and/or preferential hypertrophy of the other quadriceps femoris muscles [99] could have increased leg extension PT without affecting the MMG signal from the vastus lateralis [94,95].

Esposito et al. [96] recently examined the MMG amplitude and MPF versus isometric torque relationships for the vastus lateralis muscle of elderly men before and after an isokinetic strength training program. During this training program, the subjects performed maximal concentric isokinetic muscle actions of the dominant leg extensors at velocities of 120 and 240°·s^-1^. The training was performed twice a week for twelve weeks. The authors [96] found that the training program had no effect on the MMG amplitude values for the vastus lateralis muscle at each relative torque level (20, 40, 60, 80, and 100% MVC). At 80% and 100% MVC, however, the training program resulted in significant increases in MMG MPF for the vastus lateralis muscle. In addition, the authors [96] reported that before training, the MMG power density spectrum for the vastus lateralis during an isometric MVC was "...unimodal, with a well-defined main peak at about 11 Hz." After training, however, the MMG power density spectrum during an MVC was bimodal, with a large peak at approximately 15 Hz and a smaller peak at about 30 Hz. It was suggested [96] that the training-induced increases in MMG MPF for the vastus lateralis muscle at 80% and 100% MVC, as well as the changes in the MMG power density spectrum, may have reflected a "retrieval" of fast-twitch motor units, which are lost in some muscles during the aging process [103]. In addition, Coburn [97] recently examined the effects of three days of velocity-specific isokinetic training on the MMG amplitude and MPF values from the rectus femoris, vastus lateralis, and vastus medialis muscles. Thirty adults were randomly assigned to one of three groups: a) a control group, b) a slow velocity training group, or c) a fast velocity training group. The subjects in the two training groups performed three separate training sessions that consisted of maximal concentric isokinetic muscle actions of the nondominant leg extensors at a velocity of 30 (slow velocity training group) or 270°·s^-1 ^(fast velocity training group). In addition, the subjects in the training groups were tested for maximal concentric isokinetic leg extension PT at velocities of 30, 150, and 270°·s^-1 ^prior to, and following the training program. The subjects in the control group were also tested for leg extension PT at the same velocities, but did not perform any training. Coburn [97] found training-induced increases in leg extension PT for the fast velocity training group at 270°·s^-1 ^and for the slow velocity training group at 30, 150, and 270°·s^-1^. In addition, when compared to the control group, there were training-induced increases in MMG amplitude (averaged across the rectus femoris, vastus lateralis, and vastus medialis muscles) for the fast velocity training group at 270°·s^-1 ^and for the slow velocity training group at 150°·s^-1^. The isokinetic leg extension training did not, however, result in changes in MMG MPF for any muscle at any velocity. In addition, the training program had no effect on EMG amplitude for the rectus femoris, vastus lateralis, and vastus medialis muscles at any velocity. Thus, it was suggested that the increases in MMG amplitude for the rectus femoris, vastus lateralis, and vastus medialis muscles for the fast velocity training group at 270°·s^-1 ^and for the slow velocity training group at 150°·s^-1 ^may have been due to changes in muscle compliance. Specifically, although the EMG signal was not recorded from the hamstrings, it is possible that reduced coactivation in the biceps femoris, semitendinosus, or semimembranosus following the isokinetic training may have increased the net leg extension torque and affected compliance in the quadriceps femoris muscles [97]. As stated previously, muscle compliance could influence the muscle fiber oscillations and/or pressure waves that generate the MMG signal [71-73,86,87].

Thus, the findings from these studies [93-97] provided conflicting evidence regarding the potential effects of dynamic strength training on the amplitude and frequency contents of the MMG signal. The training-induced increases in MMG frequency for the vastus lateralis and gastrocnemius muscles reported by Cerquiglini et al. [93] and Esposito et al. [96] suggested that the frequency domain of the MMG signal may provide information regarding functional changes in skeletal muscle following a training program, such as retrieval of fast-twitch motor units in elderly individuals. In contrast, Evetovich et al. [95] found that there was no change in MMG MPF for the vastus lateralis muscle following twelve weeks of concentric isokinetic leg extension training. It is possible that the discrepancies among the results from these investigations [93,95,96] may have been due to differences in the testing methods that were used and/or the characteristics of the subjects that participated in the studies. Specifically, Evetovich et al. [95] tested subjects during maximal concentric isokinetic muscle actions, while Cerquiglini et al. [93] and Esposito et al. [96] recorded MMG signals only during isometric muscle actions. In addition, Evetovich et al. [95] tested young untrained men, while Cerquiglini et al. [93] examined Olympic weightlifters and untrained individuals, and Esposito et al. [96] investigated elderly men. Furthermore, Coburn [97] found that three days of velocity-specific isokinetic training resulted in isolated changes in MMG amplitude for the rectus femoris, vastus lateralis, and vastus medialis muscles, but Evetovich et al. [94] reported that a twelve week isokinetic resistance training program had no effect on MMG amplitude for the vastus lateralis. There are several factors that may have contributed to the differences between the results from these studies, the most obvious of which is the duration of the training program that was used (thirty-six training sessions over twelve weeks for Evetovich et al. [94] and three training sessions over approximately five to seven days for Coburn [97]). In addition, differences between the investigations in the muscles that were examined and/or the velocities that were used for the testing and training may have influenced the results for MMG amplitude. Collectively, the findings from these studies [93-97] suggested that MMG could provide information regarding the adaptations that occur during a resistance training program, but this possibility should be examined further in different muscles and/or subject groups, as well as following various types of training programs.

## Acute effects of stretching on MMG amplitude and MPF

A number of studies [104-107] have reported decreases in maximal concentric isokinetic PT following stretching. Two primary hypotheses have been proposed to explain the stretching-induced reductions in strength: a) mechanical factors, such as changes in muscle stiffness, and b) neuromuscular factors, such as altered motor control strategies and/or changes in reflex sensitivity [104,107,108]. Theoretically, both of these factors could affect MMG amplitude and MPF. For example, Barry and Cole [101] indicated that the frequency content of the MMG signal is influenced by muscle stiffness. Specifically, high levels of muscle stiffness could increase the muscle's resonant frequency, potentially resulting in a higher frequency MMG signal. In addition, several investigations [8,27,66,109] have suggested that during isometric muscle actions at high torque levels, muscle stiffness may restrict the lateral oscillations of the active muscle fibers, thereby decreasing MMG amplitude. In contrast, if stretching reduces muscle stiffness, the muscle fibers may be able to oscillate more freely and at lower frequencies. Theoretically, this could result in increases in MMG amplitude and decreases in MMG MPF. Furthermore, recent studies [4,5,10,12,14] have suggested that MMG amplitude is related to motor unit recruitment, while the MMG power density spectrum may contain information regarding the global motor unit firing rate. Thus, the time and frequency domains of the MMG signal could be useful for examining potential stretching-induced alterations in motor control strategies.

Evetovich et al. [106] examined the acute effects of static stretching of the forearm flexors on PT, MMG amplitude, and EMG amplitude during maximal concentric isokinetic muscle actions of the forearm flexors at velocities of 30 and 270°·s^-1^. The authors [106] found that the stretching resulted in a significant decrease in forearm flexion PT (averaged across the 30 and 270°·s^-1 ^velocities) and increases in MMG amplitude for the biceps brachii muscle at both velocities. The stretching had no effect, however, on the EMG amplitude values for the biceps brachii muscle at either velocity. It was suggested that the stretching-induced decreases in forearm flexion PT and increases in MMG amplitude for the biceps brachii may have been due to reduced muscle stiffness. Cramer et al. [105] examined the acute effects of static stretching of the dominant leg extensors on PT, mean power output, MMG amplitude, and EMG amplitude during maximal concentric isokinetic leg extensions at velocities of 60 and 240°·s^-1^. The authors [105] reported that the stretching resulted in decreases in PT for the stretched limb at 60 and 240°·s^-1^, as well as for the unstretched limb at 60°·s^-1^. In addition, there were stretching-induced decreases in EMG amplitude for the rectus femoris and vastus lateralis muscles for both the stretched and unstretched limbs at 60 and 240°·s^-1^. It was suggested that the decreases in leg extension PT and EMG amplitude following the static stretching may have been due, at least partially, to reduced muscle activation in the rectus femoris and vastus lateralis muscles. The stretching had no effect, however, on mean power output and MMG amplitude for the rectus femoris or vastus lateralis muscles for either limb at 60 and 240°·s^-1^. Thus, these findings provided support for the hypothesis of Bodor [44] that MMG amplitude is more closely related to power output than PT during maximal isokinetic muscle actions. Marek et al. [110] recently examined the acute effects of static and proprioceptive neuromuscular facilitation (PNF) stretching on PT, mean power output, EMG amplitude, and MMG amplitude during maximal concentric isokinetic leg extensions at velocities of 60 and 300°·s^-1^. PNF stretching is a technique that is designed to relax the stretched muscle(s) by activating Golgi tendon organs and reducing the stretch reflex, thereby allowing for less active resistance to muscle lengthening [111]. The authors [110] found that both static and PNF stretching resulted in decreases in PT, mean power output, and EMG amplitude for the rectus femoris and vastus lateralis muscles at 60 and 300°·s^-1^. In addition, there was an increase in MMG amplitude following the static stretching, but only for the rectus femoris muscle at 60°·s^-1^. It was suggested that the stretching-induced decreases in PT, mean power output, and EMG amplitude for the rectus femoris and vastus lateralis may have been due to a combination of reduced muscle activation and decreases in muscle stiffness.

The acute effects of static stretching have also been examined during maximal eccentric isokinetic muscle actions. Eccentric muscle actions may provide an interesting situation for examining potential stretching-induced changes in PT and MMG amplitude, because Wilson et al. [112] found that unlike concentric and isometric muscle actions, maximal eccentric torque was not related to musculotendonous stiffness. Thus, decreases in muscle stiffness following stretching could, theoretically, result in increases in MMG amplitude, with no change in eccentric PT. Cramer [113] examined the acute effects of static stretching on PT, mean power output, EMG amplitude, EMG MPF, MMG amplitude, and MMG MPF during maximal eccentric isokinetic muscle actions of the leg extensors at velocities of 60 and 180°·s^-1^. The results indicated that there were no meaningful changes in PT, EMG amplitude, EMG MPF, and MMG MPF following the static stretching. There were, however, stretching-induced decreases in mean power output and MMG amplitude for the rectus femoris muscle at 60 and 180°·s^-1^. It was suggested that the decreases in MMG amplitude for the rectus femoris muscle following stretching may have been due to a close relationship between MMG amplitude and mean power output.

Collectively, the results from these studies [105,106,110,113] indicated that the acute effects of stretching on MMG amplitude are muscle-specific as well as specific to the type of muscle action. For example, during maximal concentric isokinetic muscle actions, static stretching resulted in an increase in MMG amplitude for the biceps brachii [106], but not for the rectus femoris and vastus lateralis [105]. In addition, during maximal eccentric isokinetic muscle actions, static stretching actually decreased MMG amplitude for the rectus femoris and vastus lateralis [113]. The results from these studies also provided support for the hypothesis of Bodor [44] that MMG amplitude is more closely related to power output than PT during maximal isokinetic muscle actions. Furthermore, the stretching-induced decreases in PT and EMG amplitude in both the stretched and unstretched limbs during maximal concentric isokinetic muscle actions [105] suggested that decreases in PT following static stretching may be partially due to neuromuscular factors such as altered motor control strategies and/or changes in reflex sensitivity. Thus, simultaneous examination of EMG and MMG may provide information regarding the relative contributions of neural (EMG) and mechanical (MMG) factors to stretching-induced decreases in isokinetic PT.

## Processing MMG signals recorded during dynamic muscle actions

During voluntary isometric muscle actions, the MMG signal is generated by a nonlinear summation of the mechanical activities from the unfused activated motor units [4-6,114] and, in most cases, various sources of noise (e.g. movement artifact created by displacement of the muscle and/or limb) do not have a large influence on MMG amplitude or frequency. This is not always the case during dynamic muscle actions. For example, movement artifact is often present in biological signals such as the surface EMG [115-117], electroencephalogram (EEG) [118], and electrocardiogram (ECG) [119], and it has been suggested [120-122] that large limb and/or muscle movements may also influence the MMG signal. In addition, changes in muscle length and the thickness of the tissue between the muscle and the MMG sensor could affect MMG amplitude and frequency during a dynamic muscle action [1,2,25]. Thus, the MMG signals recorded during dynamic activities are typically nonstationary [123] and may require different signal processing methodologies when compared to isometric muscle actions. Specifically, there are three factors that are particularly important when analyzing MMG signals recorded during dynamic muscle actions: a) filtering the signal to attenuate various sources of noise, b) choosing the length of the epoch(s) (i.e. window) to select from the recorded signal, and c) using the appropriate method to analyze the signal's frequency content.

**Figure 7:**
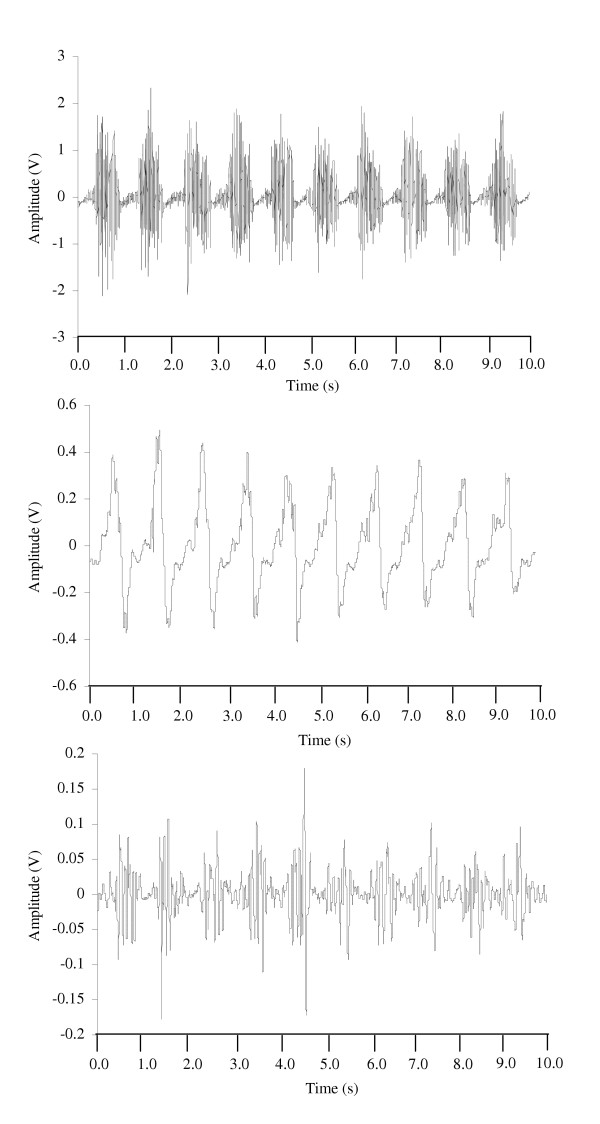
**Attenuation of movement artifact with digital filtering**. The *top graph *demonstrates a ten second sample of the unfiltered electromyographic (EMG) signal from the rectus femoris muscle during cycle ergometry at a power output of 230 W. The *middle graph *shows the corresponding unfiltered mechanomyographic (MMG) signal from the rectus femoris muscle. The *bottom graph *demonstrates the MMG signal from the rectus femoris muscle after digital filtering with a zero lag, fourth-order Butterworth bandpass filter with cutoff frequencies of 5 and 100 Hz. The MMG signal was detected with a contact sensor (HP 21050A) and sampled at 1,000 Hz.

One of the most important considerations in filtering MMG signals is selecting the appropriate cutoff frequencies. Most previous studies have used analog and/or digital bandpass filters with cutoff frequencies between 1 and 250 Hz [9,68,124-130] when processing MMG signals recorded during isometric muscle actions. Other investigations, however, have used filters with different cutoff frequencies to reduce various types of noise in the MMG signal. For example, Mealing et al. [84] used a 3 Hz high pass filter to attenuate arterial sounds, while Goldenberg et al. [83] used a high pass filter with a cutoff frequency of 14 Hz to reduce the influence of tremor on the MMG signal. In addition, Weir et al. [131] used a 60 Hz notch filter to remove potential powerline interference from the MMG signal. Filters have also been used to isolate specific frequency components of the MMG power density spectrum. For example, Petitjean and Bellemare [132] used a 30 Hz high pass filter to examine the high frequency components of MMG signals from the diaphragm muscle. It was suggested [132] that the filtered signals may provide information regarding the resonant frequency of the muscle being investigated [101]. During dynamic muscle actions, however, filters have been used primarily to attenuate movement artifact (see Figure 7). Movement artifact is a source of low frequency noise in many biological signals [115-119]. For high frequency signals, filtering the movement-related noise usually results in less attenuation of the original signal because the major portions of the spectra from the noise and the signal of interest do not overlap substantially [133]. For example, the influence of movement artifact is often reduced in surface EMG signals by high pass filtering with a cutoff frequency between 20 and 30 Hz [115]. In most cases, this does not severely attenuate the original EMG signal because most of the power in surface EMG is between approximately 40 and 160 Hz [134]. For lower frequency signals (such as MMG), however, the low frequency cutoff should be carefully selected to attenuate as much noise as possible, yet preserve the signal of interest. Petitjean et al. [64] used a bandpass filter with cutoff frequencies of 10 and 60 Hz to remove noise from MMG signals recorded during concentric DCER muscle actions of the biceps brachii and brachioradialis. Dalton and Stokes [26] used a bandpass filter with cutoff frequencies of 8 and 160 Hz when processing MMG signals from the biceps brachii recorded during concentric and eccentric DCER muscle actions of the forearm flexors. Most studies, however, have used a filter with a 5 Hz high pass cutoff frequency to attenuate movement artifact in MMG signals [120-122]. Theoretically, this technique reduces the influences of body and respiratory movements, as well as gross limb displacements [122].

Another important factor in processing MMG signals recorded during dynamic muscle actions is choosing the length of the epoch(s) that will be selected from the recorded signal. For example, several studies from our laboratory have examined the MMG responses from various muscles during maximal concentric isokinetic muscle actions. Concentric isokinetic exercise involves three main phases of movement: a) acceleration, b) constant velocity, and c) deceleration [135]. The constant velocity portion of the movement is often referred to as the "load range" and is characterized by a match between isokinetic velocity and limb movement [135]. During the acceleration and deceleration phases of the movement, however, there is no quantifiable external load because the limb is accelerating or decelerating [135]. Therefore, during the acceleration and deceleration phases of movement, changes in velocity could potentially affect the amplitude and/or frequency contents of the MMG signal. Although this phenomenon may be interesting in itself, it is important to examine the MMG signal during the constant velocity portion of the movement (i.e. load range) if the potential effect(s) of changes in movement speed is to be avoided. The technique used most often in our laboratory is to select the MMG signal from the middle third of the range of motion [35,37,48,71,95]. For example, during a concentric isokinetic leg extension performed across a 90° range of motion, the MMG signal is selected from approximately 120° to 150° of leg extension [35]. This procedure has been used successfully for velocities ranging from 30 to 360°·s^-1 ^and allows the MMG signal to be examined across a standardized range of motion [35,37]. Alternatively, if the dynamometer provides velocity data, then the MMG signal can be selected when the constant velocity portion of the movement is achieved.

Another important consideration in processing MMG signals recorded during dynamic muscle actions is selecting the appropriate method for analyzing the signal's frequency content. Most previous studies have used Fourier-based algorithms, such as the fast Fourier transform (FFT) or discrete Fourier transform (DFT) [27,43,66,121,136], although several investigations have also used the maximum entropy spectrum estimation (MESE) [5,9,76,137] or autocorrelation [138] techniques. Perhaps the primary factor in determining which method to use for frequency analysis is whether or not the signal being analyzed is stationary. In particular, the FFT and DFT assume that the input signal is stationary, and according to Bonato et al. [139], wide-sense stationarity can be assumed for surface EMG signal epochs between 0.5 and 2.0 seconds during isometric muscle actions at constant force levels. Assuming that similar conditions must be met for the MMG signal to be considered wide-sense stationary, the MMG signal may indeed be nonstationary during dynamic muscle actions. There are several factors that could potentially influence the amplitude and/or frequency contents (and the subsequent stationarity) of the MMG signal during a dynamic muscle action. For example, Barry [1] reported that during electrically-stimulated isometric twitches of isolated frog gastrocnemius muscle, the amplitude of the MMG signal increased with decreases in muscle length and was greatest when the muscle was held at approximately 90% of the optimal length for force production. Frangioni et al. [2] also examined isolated frog gastrocnemius muscle during electrically stimulated twitches and found that the "characteristic frequency" (MPF divided by the area under the power density spectrum) of the MMG signal decreased when the muscle was held at shorter lengths. Thus, changes in muscle length during a dynamic muscle action could potentially influence MMG amplitude and/or frequency, resulting in a nonstationary MMG signal. Furthermore, changes in the thickness of the tissue between the muscle and the MMG sensor may also affect MMG amplitude and frequency. As stated previously, the tissue between the muscle and the MMG sensor acts as a low pass filter that attenuates the muscle fiber oscillations and pressure waves that generate the MMG signal [4,25]. Jaskólska et al. [25] suggested that the filtering effect of this layer becomes greater with increases in tissue thickness. Thus, changes in the thickness of the tissue between the muscle and the MMG sensor during a dynamic muscle action could influence the amplitude and/or frequency contents of the MMG signal, potentially affecting signal stationarity.

**Figure 8:**
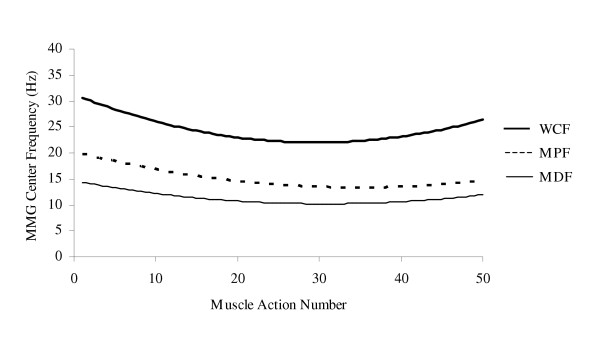
**Wavelet- vs. Fourier-based estimates of mechanomyographic (MMG) center frequency**. Example of the relationships for mechanomyographic (MMG) center frequencies [wavelet center frequency (WCF), mean power frequency (MPF), and median frequency (MDF)] for the biceps brachii muscle of one subject during 50 consecutive maximal concentric isokinetic muscle actions of the forearm flexors at a velocity of 180°·s^-1^. The relationships shown are the best-fit regression models for the MMG WCF, MPF, and MDF data. The discrete wavelet transform was used to calculate the MMG WCF values, and the fast Fourier transform was used to obtain the MMG MPF and MDF values.

Despite the limitations of using Fourier-based methods to analyze nonstationary MMG signals, recent studies [123,140,141] have found that the discrete wavelet transform (DWT) and continuous wavelet transform (CWT) (which do not assume signal stationarity) provided very similar results when compared to the FFT. For example, Beck et al. [123] reported that during 50 consecutive maximal concentric isokinetic muscle actions of the forearm flexors at a velocity of 180°·s^-1^, the FFT and DWT resulted in quadratic decreases in MMG center frequencies (MPF and median frequency for the FFT and wavelet center frequency for the DWT) for the biceps brachii. In addition, the normalized MMG MPF, median frequency, and wavelet center frequency values for the biceps brachii were significantly intercorrelated (r = 0.671–0.935) [123]. Similar results were found when comparing Fourier- and wavelet-based methods for processing MMG signals recorded during submaximal to maximal concentric [140] and eccentric [141] isokinetic muscle actions of the forearm flexors. Thus, these findings suggested that despite the assumption of signal stationarity, Fourier-based methods are acceptable for determining the patterns for MMG center frequency during dynamic muscle actions (see Figure 8).

In addition to the potential confounding factors that may influence the MMG signal during dynamic muscle actions, changes in recruitment and/or firing rate could also affect MMG amplitude and frequency. For example, Theeuwen et al. [53] reported that during concentric muscle actions of the forearm flexors performed against an elastic load (i.e. torque production increased as the angle between the arm and the forearm decreased), there was an increase in the number of active motor units in the biceps brachii (assessed with intramuscular EMG electrodes) as the muscle shortened and produced greater torque. Furthermore, Kossev and Christova [142] suggested that during eccentric muscle actions of the forearm flexors, firing rate modulation may be important in the biceps brachii for changing torque production across the range of motion. As stated previously, MMG amplitude could be closely related to recruitment [12], while the MMG power density spectrum may provide qualitative information regarding the global firing rates of the unfused activated motor units [5,13,14]. Thus, changes in MMG amplitude and frequency across the range of motion during a dynamic muscle action could reflect motor unit recruitment and/or firing rate modulation.

**Figure 9:**
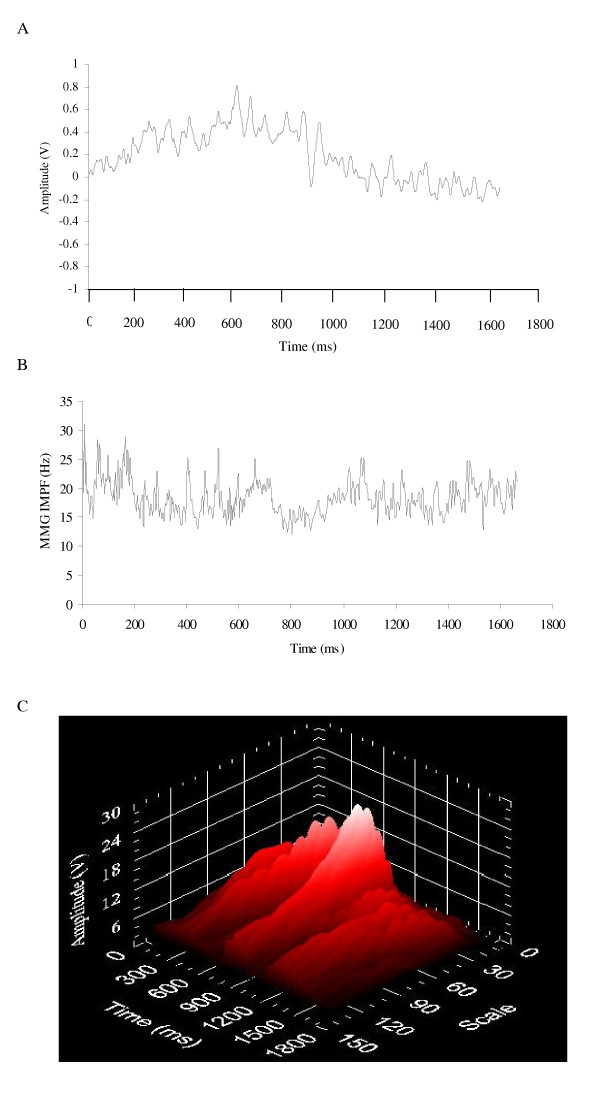
**Joint time-frequency analysis of a mechanomyographic (MMG) signal recorded during a concentric isokinetic muscle action**. (A) Example of the raw mechanomyographic (MMG) signal from the biceps brachii muscle during a maximal concentric isokinetic muscle action of the forearm flexors at a velocity of 30°·s^-1^. The MMG signal was detected by a contact sensor (HP 21050A) and was selected from the middle 50° of the range of motion (approximately 110° to 160° of forearm flexion). (B) The relationship between MMG instantaneous mean power frequency (IMPF) and time for the MMG signal shown in (A). The MMG signal was processed with the continuous wavelet transform (CWT) algorithm and a Daubechies 10 wavelet with a center frequency of 684.2 Hz at the lowest scale. (C) The scalogram of the signal shown in (A) demonstrates the changes in the signal's frequency content across the range of motion.

**Figure 10:**
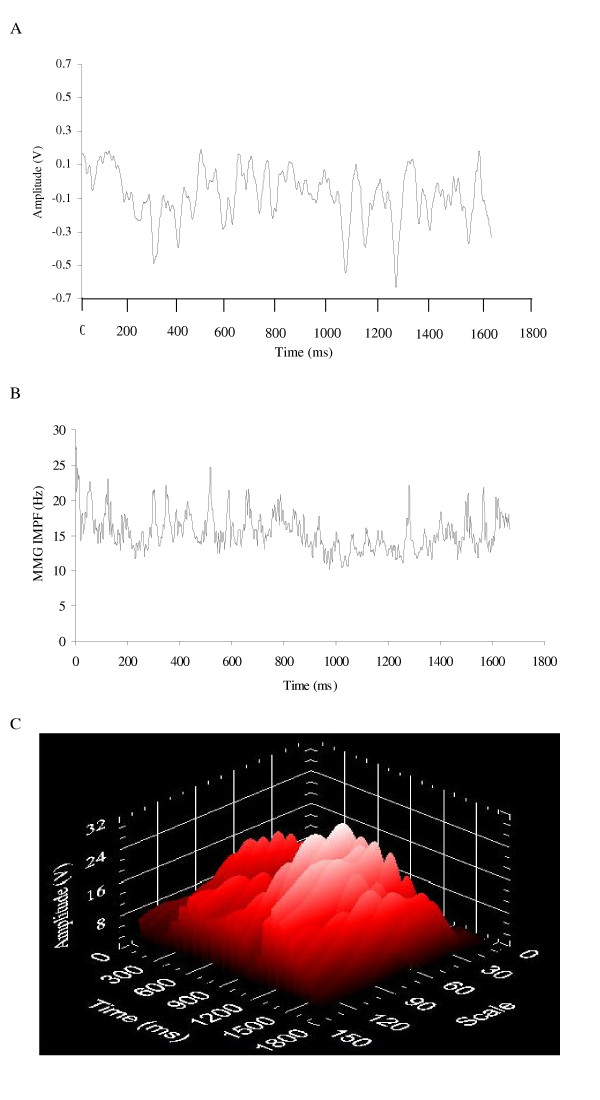
**Joint time-frequency analysis of a mechanomyographic (MMG) signal recorded during an eccentric isokinetic muscle action**. (A) Example of the raw mechanomyographic (MMG) signal from the biceps brachii muscle during a maximal eccentric isokinetic muscle action of the forearm flexors at a velocity of 30°·s^-1^. The MMG signal was detected by a contact sensor (HP 21050A) and was selected from the middle 50° of the range of motion (approximately 110° to 160° of forearm flexion). (B) The relationship between MMG instantaneous mean power frequency (IMPF) and time for the MMG signal shown in (A). The MMG signal was processed with the continuous wavelet transform (CWT) algorithm and a Daubechies 10 wavelet with a center frequency of 684.2 Hz at the lowest scale. (C) The scalogram of the signal shown in (A) demonstrates the changes in the signal's frequency content across the range of motion.

Traditional Fourier-based methods, such as the FFT and DFT, provide information only in the frequency domain (i.e. all time domain information is lost). Thus, these techniques are limited in terms of their ability to track rapid changes in the frequency content of the input signal. Joint time-frequency methods, such as the Wigner transform and CWT, however, provide information in both the time and frequency domains, and, therefore, are particularly useful for analyzing nonstationary signals (see Figures 9 and 10). For example, Barry and Cole [101] used the Wigner transform to examine the patterns for MMG MPF and peak frequency from isolated frog gastrocnemius muscle during an electrically-stimulated tetanic contraction. It was suggested that alterations in MMG peak frequency during the tetanus may have reflected changes in the resonant frequency of the muscle. In addition, Karlsson et al. [143] used the CWT to examine the patterns for EMG instantaneous mean power frequency (IMPF) and instantaneous magnitude (IM) across the range of motion during maximal concentric isokinetic leg extensions at velocities ranging from 0 to 180°·s^-1^. The surface EMG signals were recorded from the rectus femoris and vastus lateralis muscles. Karlsson et al. [143] suggested that increases in EMG IMPF and IM across the range of motion during a maximal concentric isokinetic muscle action may "...reflect the successive recruitment of MUs [motor units]." Although no previous studies have examined the patterns for MMG amplitude and/or MPF across the range of motion during a dynamic muscle action, the advent of joint time-frequency methods has improved the ability to analyze signals with rapidly changing frequency contents. Future investigations, in which techniques such as the Wigner transform or CWT are applied to MMG signals recorded during dynamic muscle actions, may provide information regarding the motor control strategies that are used to increase or decrease torque production across the range of motion.

Collectively, the results from these studies indicated that in some cases, MMG signals recorded during dynamic muscle actions could require different signal processing techniques when compared to those during isometric muscle actions. Specifically, a high pass filter with a cutoff frequency of approximately 5 Hz may be necessary to attenuate the potential influence(s) of movement artifact on the MMG signal [120-122]. In addition, during concentric isokinetic muscle actions, the MMG signal should be analyzed during the constant velocity portion of the movement (i.e. load range) to avoid the acceleration and deceleration phases [135]. Several studies [123,140,141] have also provided evidence that despite the assumption of signal stationarity, traditional Fourier-based methods (such as the FFT or DFT) are acceptable for examining the patterns of response for MMG center frequency during dynamic activities, although joint time-frequency methods are required to examine potential changes in MMG frequency across the range of motion during a dynamic muscle action.

## Miscellaneous

Although many studies have examined the potential for MMG to be used as a tool for investigating motor control strategies, several studies have also used MMG to examine various aspects of muscle function, including post-exercise muscle soreness [120], phonomechanical delay [64], energy requirements during concentric, isometric, and eccentric muscle actions [144], excess post-exercise oxygen consumption (EPOC) [145], dehydration [146], and hyperhydration [147]. In addition, Beck et al. [148] recently compared a contact sensor (Hewlett-Packard 21050A) with an accelerometer (Entran EGAS FT 10) for examining the MMG amplitude and MPF versus torque relationships for the biceps brachii during isometric and concentric isokinetic muscle actions of the forearm flexors at a velocity of 30°·s^-1^. The authors [148] found that during both the isometric and concentric isokinetic muscle actions, the contact sensor and accelerometer resulted in linear increases in normalized MMG amplitude with torque, but the linear slope of the normalized MMG amplitude versus isokinetic torque relationship for the accelerometer was less than that of the contact sensor. Furthermore, there were no significant relationships for normalized MMG MPF versus isokinetic and isometric torque for the contact sensor, but the accelerometer demonstrated a quadratic or linear relationship for the isokinetic and isometric muscle actions, respectively. The authors [148] suggested that in some cases involving dynamic and isometric muscle actions, a contact sensor and accelerometer may result in different torque-related patterns for MMG amplitude and/or MPF, thereby affecting the interpretation of the motor control strategies involved.

Bajaj et al. [120] investigated the MMG amplitude responses for the first dorsal interosseous during a series of concentric, isometric, and eccentric muscle actions. For each series of muscle actions, the subjects performed a single abduction movement of the index finger (concentric muscle action), followed immediately by a 2-sec isometric muscle action of the first dorsal interosseous with the index finger in the fully abducted position, and then a single adduction movement of the index finger (eccentric muscle action). These muscle actions were performed at four different relative torque levels (0, 25, 50, 75, and 100% of the isometric MVC) prior to, immediately following, and 24 and 48 hours after a series of maximal eccentric muscle actions of the first dorsal interosseous. The authors [120] found that the MMG amplitude values for the first dorsal interosseous (averaged across the type of muscle action and percentage of the MVC) immediately after the eccentric exercise were greater than the values recorded prior to exercise and following 24 and 48 hours of rest. It was suggested that the increase in MMG amplitude immediately after the eccentric exercise may have been due to greater physiological tremor and/or increased edema in the muscle fibers of the first dorsal interosseous. In addition, the authors [120] reported increased subjective pain ratings immediately after the eccentric exercise. It was hypothesized [120] that the eccentric exercise may have allowed for greater muscle fiber vibrations that resulted in activation of "mechanosensitive deep tissue nociceptors." Theoretically, activation of these receptors could have contributed to the increased pain sensations felt immediately after the eccentric exercise.

Petitjean et al. [64] examined "phonomechanical delay" (the time interval between the onsets of the MMG and acceleration signals) in the biceps brachii and brachioradialis during submaximal concentric DCER muscle actions of the forearm flexors performed against a 3-kg weight. The concentric muscle actions involved accelerating the weight at slow (20–120 rad·s^-2^), intermediate (120–240 rad·s^-2^), and fast (240–360 rad·s^-2^) angular velocities. The authors [64] found that phonomechanical delay for the biceps brachii and brachioradialis muscles increased with acceleration, and it was hypothesized that the onsets of the MMG signals may have reflected development of tension in the contractile components of the muscles. In addition, the phonomechanical delay values were greater than those from previous studies during supramaximal electrical stimulation of isolated frog gastrocnemius muscle [1], and intact tibialis anterior [149] and thenar muscle [7]. Petitjean et al. [64] suggested that the greater phonomechanical delay values for the biceps brachii and brachioradialis may have been due to differences between submaximal voluntary and supramaximal electrically stimulated muscle actions. Specifically, during submaximal voluntary muscle actions, high threshold fast-twitch motor units with short contraction times may not be activated. During supramaximal electrical stimulation, however, all muscle fibers are activated simultaneously, and, therefore, tension may be developed more rapidly in the muscle [64].

Vedsted et al. [144] recently examined the MMG amplitude responses for the biceps brachii during concentric, isometric, and eccentric DCER muscle actions of the forearm flexors at 10% and 20% of the isometric MVC. Muscle tissue oxygenation was also measured from the biceps brachii to provide information regarding the energy requirements during concentric, isometric, and eccentric muscle actions. The authors [144] found that the MMG amplitude values for the biceps brachii were greater during the concentric and eccentric muscle actions than during the isometric muscle actions. There were no differences, however, among the muscle tissue oxygenation levels for the biceps brachii during the concentric, isometric, and eccentric muscle actions. It was suggested [144] that during the concentric and eccentric muscle actions, low motor unit firing rates in the biceps brachii could have resulted in less fusion of motor unit twitches and a "...more distinct mechanical twitching..." that allowed larger muscle fiber oscillations and greater MMG amplitude values. During the isometric muscle actions, however, high motor unit firing rates may have resulted in greater fusion of motor unit twitches and reduced MMG amplitude values. The authors [144] also suggested that selective recruitment of superficially located fast-twitch motor units, particularly during the eccentric muscle actions, may have allowed the active motor units to "...influence more directly the MMG generation process." Theoretically, this could also result in greater MMG amplitude values.

McKay et al. [145] examined the responses for MMG amplitude, MMG MPF, and MMG peak frequency for the rectus femoris muscle prior to, and immediately following a 30-min cycle ergometer workbout that was performed at a power output that corresponded to 70% of the maximal power output achieved during a VO_2_ max test. The authors [145] reported that immediately after the cycle ergometer workbout, the VO_2_ and MMG amplitude values for the rectus femoris muscle were greater than the respective values recorded prior to exercise, but there were no differences between the pre- and post-exercise values for MMG MPF or peak frequency. In addition, MMG amplitude and VO_2_ decreased exponentially during a 5.5-h recovery period after exercise. It was hypothesized [145] that the increased MMG amplitude and VO_2_ values after exercise may have been due to greater motor unit mechanical activities and oxygen utilization as the quadriceps femoris muscles returned to the pre-exercise state. In addition, the similar decreases in MMG amplitude and VO_2_ during the recovery period after exercise suggested that the mechanical (MMG) and metabolic (VO_2_) aspects of recovery may be closely related.

Recent investigations have also examined the effects of dehydration [146] and hyperhydration [147] on MMG amplitude and MPF. Several studies [35,37,38,43,57] have suggested that the MMG signal may be influenced, at least partially, by movement of the fluid that surrounds muscle fibers. Thus, experimentally-induced alterations in the intracellular and extracellular fluid media (i.e. through dehydration or hyperhydration) could potentially affect the amplitude and frequency contents of the MMG signal. Evetovich et al. [146,147] examined the effects of dehydration and hyperhydration on maximal concentric isokinetic forearm flexion PT, MMG amplitude, and MMG MPF for the biceps brachii muscle. Isokinetic PT was measured at a velocity of 90°·s^-1^, and dehydration was achieved through restriction of water intake. The subjects were considered dehydrated if there was a change in body weight of > 2.0% and the urine specific gravity was > 1.020 [146]. Hyperhydration, however, was achieved through ingestion of glycerol, which results in fluid retention in all water compartments of the body. The hyperhydration state was ensured by examining changes in body weight following glycerol ingestion, as well as measuring the percent fluid retention [(volume of fluid consumed - urine volume voided)/volume of fluid consumed] [147]. The authors [146,147] found that neither dehydration nor hyperhydration had any effect on isokinetic PT, MMG amplitude, or MMG MPF. It was suggested that the MMG signal may be influenced more by motor control strategies and the intrinsic contractile properties of muscle than by the fluids that surround muscle fibers.

Collectively, the results from these studies [64,120,144-148] indicated that MMG amplitude and MPF responses may be influenced by the type of sensor that is used to detect the signal, and MMG could be useful for examining various aspects of muscle function during dynamic muscle actions. Specifically, Beck et al. [148] found that in some cases, a contact sensor and accelerometer resulted in different patterns for MMG amplitude and MPF versus isokinetic and isometric torque. Thus, the type of sensor that is used to detect the MMG signal could affect the interpretation of the motor control strategies involved. Bajaj et al. [120] reported increased MMG amplitude values for the first dorsal interosseous muscle immediately following eccentric exercise, and suggested that MMG could potentially be used to examine the mechanisms that underlie post-exercise muscle soreness. In addition, Petitjean et al. [64] found that phonomechanical delay in the biceps brachii and brachioradialis during concentric DCER muscle actions of the forearm flexors was dependent on the rate of acceleration. It was hypothesized that the onsets of the MMG signals may have reflected development of tension in the contractile components of the muscles. Vedsted et al. [144] reported that the MMG amplitude values for the biceps brachii were greater during concentric and eccentric DCER muscle actions of the forearm flexors than during isometric muscle actions at the same absolute torque level. It was suggested that when compared to isometric muscle actions, lower motor unit firing rates during concentric and eccentric muscle actions may have resulted in less fusion of motor unit twitches and greater MMG amplitude values. McKay et al. [145] found that VO_2_ and MMG amplitude for the rectus femoris muscle were elevated immediately after a 30-min cycle ergometer workbout, but both parameters (VO_2_ and MMG amplitude) decreased exponentially as the quadriceps femoris muscles returned to the resting state. It was suggested that in the resting state, MMG may be closely related to oxygen utilization. Finally, Evetovich et al. [146,147] found that dehydration and hyperhydration had no effect on MMG amplitude and MPF for the biceps brachii muscle. It was hypothesized that the MMG signal may be influenced primarily by motor control strategies and the intrinsic contractile properties of muscle than by the fluids that surround muscle fibers. Thus, the findings from these studies [64,120,144-148] supported the use of MMG for examining various aspects of muscle function. Future studies should continue investigating the unique applications of MMG amplitude and frequency responses with different experimental designs/methodologies to continually reassess the uses/limitations of MMG.

## Summary, conclusions, and future research

Each of the studies that were examined in the present review has made an important contribution to MMG research. Although MMG is growing in popularity in fields such as biomechanics, exercise physiology, biomedical engineering, and medicine, the literature base for MMG is probably 20–25 years behind that of EMG. Thus, it is important to continually examine the potential uses/applications of MMG in a variety of experimental situations, including dynamic activities [6]. During dynamic muscle actions, many factors such as changes in torque production, muscle length, the thickness of the tissue between the muscle and the MMG sensor, motor unit recruitment, and firing rate can influence the amplitude and frequency of the MMG signal. These factors add to a list of mechanisms that may affect the MMG signal, which is considered to be a complex signal [150], even during isometric muscle actions. Thus, dynamic muscle actions are often avoided in MMG research on the grounds that during these activities, there are too many confounding factors that could influence the MMG signal and render the resulting data uninterpretable and unusable. There is substantial evidence, however, to suggest otherwise.

For example, Shinohara et al. [30] reported that MMG amplitude for the vastus lateralis muscle increased linearly with power output during incremental cycle ergometry, but there was only a small increase in MMG amplitude during passive cycling in which one of the investigators pushed the pedals of the cycle ergometer. In addition, Cramer et al. [48] found that during maximal concentric and eccentric isokinetic muscle actions of the leg extensors at velocities of 60°·s^-1^, the potential for cross-talk in MMG signals was relatively small, even for muscles that are close to each other and have a common innervation. Thus, these findings indicated that during dynamic muscle actions, the MMG signal is generated primarily by muscle activity, and the characteristics of the signal are specific to the muscle(s) being examined. This is of premier importance if MMG can be used to provide valid information regarding muscle function during dynamic activities. Furthermore, MMG responses are also influenced by the type of dynamic muscle action that is being performed (concentric versus eccentric), as well as the velocity of the movement, which provides further support for the argument that during dynamic activities, the MMG signal provides meaningful information regarding muscle function. For the sake of brevity, this section will summarize only the major findings from the investigations that were examined in this review. The potential applications of MMG research will also be addressed.

### MMG amplitude and power output

One of the most consistent findings among the studies that have examined the MMG signal during dynamic muscle actions is the close relationship between MMG amplitude and power output. This has been demonstrated during both concentric [45] and eccentric [59] isokinetic muscle actions, as well as during incremental cycle ergometry [29-32]. Thus, these studies provided support for the hypothesis of Bodor [44] that MMG amplitude may be more closely related to power output than PT during maximal isokinetic muscle actions. A potentially useful application of this phenomenon is in sports medicine and/or rehabilitation, where MMG amplitude may provide information regarding training-induced increases in muscle power output. Future studies should test this hypothesis, however, in various muscles and under controlled experimental conditions.

### Linear relationship between MMG amplitude and torque

Another particularly interesting finding is the highly linear relationship between MMG amplitude and torque during concentric isokinetic and DCER muscle actions. This has been reported for both the biceps brachii [26,27,65] and vastus medialis [66,69] muscles, and is unlike the MMG amplitude versus isometric torque relationship for most large limb muscles (such as the biceps brachii and superficial quadriceps femoris muscles), in which MMG amplitude often increases from 0% to approximately 60–80% MVC and then plateaus or decreases at higher torque levels [5,8,10,109]. The linearity of the relationship between MMG amplitude and concentric torque suggests, however, that it could potentially be used to develop a mechanical analog to the "efficiency of electrical activity" (EEA) procedure of deVries [151]. Specifically, deVries [151] proposed that a decrease in the linear slope coefficient for the EMG amplitude versus isometric torque relationship indicated a reduction in the electrical activity that was required to produce a given level of torque and an improvement in muscle function. The EEA technique has been used to examine issues such as the time course of neural versus hypertrophic contributions to training-induced strength gains and the mechanisms underlying the cross-training effect [151]. The validity of the EEA procedure is dependent, however, on a linear EMG amplitude versus isometric torque relationship with a reliable slope coefficient [152]. Thus, future studies should examine the linearity and reliability of the MMG amplitude versus concentric torque relationship in different types of muscles and subjects prior to applying the EEA methodology to MMG.

### MMG signal during fatigue

Furthermore, there is evidence to suggest that the time and frequency domains of the MMG signal may provide information regarding the motor control strategies that are used during fatiguing dynamic muscle actions. For example, Beck et al. [72] reported decreases in both MMG amplitude and MMG MPF for the biceps brachii during 50 consecutive maximal concentric isokinetic muscle actions of the forearm flexors at a velocity of 180°·s^-1^. Perry-Rana et al. [71] also reported reductions in MMG amplitude for the rectus femoris, vastus lateralis, and vastus medialis during fatiguing maximal concentric isokinetic muscle actions of the leg extensors at velocities of 60, 180 and 300°·s^-1^. It was suggested [71,72] that the decreases in MMG amplitude and MPF may have been due to fatigue-related reductions in motor unit firing rates (i.e. muscle wisdom) and/or de-recruitment of fast-twitch motor units. In addition, the MMG amplitude responses during continuous cycle ergometry performed at constant, submaximal power outputs were dependent upon the power output at which the workbout was performed, as well as the muscle that was being examined [85,87]. Specifically, MMG amplitude for the vastus lateralis muscle decreased during continuous cycle ergometry at power outputs below 65% Wpeak[85,87]. At 80% Wpeak, MMG amplitude remained stable for both the vastus medialis and vastus lateralis muscles, and at 95% Wpeak, MMG amplitude increased for the vastus medialis, but did not change for the vastus lateralis [87]. It was suggested that decreases in MMG amplitude for power outputs below 65% Wpeak may have been due to fatigue-induced reductions in motor unit firing rates. In addition, the lack of a significant change in MMG amplitude at 80% Wpeak could have reflected a balance between the influences of recruitment (which can increase MMG amplitude) and decreases in motor unit firing rates (which can reduce MMG amplitude). Finally, the increases in MMG amplitude for the vastus medialis muscle at 95% Wpeak suggested that recruitment may have had a greater influence on MMG amplitude than potential fatigue-induced decreases in motor unit firing rates. Thus, the results from these studies [71,72,85,87] suggested that MMG could potentially be useful in clinical and rehabilitative settings for examining the mechanisms that underlie neuromuscular fatigue. Furthermore, future studies should consider examining the MMG MPF responses during continuous, constant power output cycle ergometry. Theoretically, MMG MPF could provide information regarding changes in the global motor unit firing rate during fatiguing cycle ergometry. It may also be beneficial to examine the potential relationships between MMG amplitude or MPF and power output during fatiguing exercise.

### MMG signal reflective of chronic and acute muscle adaptations

There is also evidence that the time and frequency domains of the MMG signal may provide information regarding the adaptations that occur during training programs and the acute effects of stretching on various aspects of muscle function. For example, Cerquiglini et al. [93] and Esposito et al. [96] reported increases in MMG frequency for the vastus lateralis and gastrocnemius muscles following a dynamic resistance training program, which suggested that the frequency domain of the MMG signal could be useful for examining the mechanisms that underlie training-induced increases in muscle strength. In addition, Evetovich et al. [106] found increases in MMG amplitude for the biceps brachii following static stretching of the forearm flexors, but there was no change in EMG amplitude. In contrast, Cramer et al. [105] reported that static stretching of the leg extensors had no effect on MMG amplitude for the rectus femoris and vastus lateralis, but there were stretching-induced decreases in EMG amplitude for both muscles, as well as in the unstretched limb. Both studies [105,106], however, reported decreases in maximal concentric isokinetic PT following static stretching. Thus, these findings suggested that simultaneous examination of EMG and MMG may provide information regarding the relative contributions of neural (EMG) and mechanical (MMG) factors to stretching-induced decreases in concentric isokinetic PT.

### Signal processing techniques for dynamic MMG

In addition, MMG signals recorded during dynamic muscle actions could require different signal processing methodologies when compared to isometric muscle actions. Specifically, a high pass filter with a cutoff frequency of approximately 5 Hz may be necessary to attenuate the potential influence of movement artifact on MMG amplitude and frequency. During isokinetic muscle actions, the MMG signal should be selected from the constant velocity portion of the movement (i.e. load range) to avoid the potential influence(s) of changes in velocity during the acceleration and deceleration phases. In addition, the advent of joint time-frequency methods, such as the Wigner transform and CWT, has improved the ability to analyze nonstationary signals. Future studies, in which the Wigner transform or CWT are applied to MMG signals, may provide information regarding the motor control strategies that modulate torque production across the range of motion during a dynamic muscle action.

Thus, the studies that were examined in this review have extended MMG research beyond the original investigations of isometric muscle actions. Both dynamic and isometric muscle actions have unique characteristics that may affect the MMG signal differently. Furthermore, the literature base for MMG during dynamic as well as isometric muscle actions is far from complete. In fact, there are still many questions regarding the exact origins of the MMG signal. Despite the differences between dynamic and isometric muscle actions, it is apparent that MMG can be used in both situations to provide valid information regarding muscle function. Thus, these characteristics refute the notion that during dynamic muscle actions, the MMG signal is too complex to provide any meaningful information. It is important for future research to continue examining MMG amplitude and frequency responses during both dynamic and isometric muscle actions in an effort to fully assess the potential uses/applications of MMG.

## Delcaration of Competing interests

The author(s) declare that they have no competing interests.

## Authors' contributions

TWB was the primary author for the manuscript. TJH carried out the majority of the editing. JTC, JPW, and JWC helped create the figures and performed some editing. GOJ, MHM, and MM assisted with formatting the manuscript and editing. All authors read and approved the final manuscript.
